# Metabolic Impacts of Using Nitrogen and Copper-Regulated Promoters to Regulate Gene Expression in *Neurospora crassa*

**DOI:** 10.1534/g3.115.020073

**Published:** 2015-07-20

**Authors:** Shouqiang Ouyang, Consuelo N. Beecher, Kang Wang, Cynthia K. Larive, Katherine A. Borkovich

**Affiliations:** * Department of Plant Pathology and Microbiology, University of California, Riverside, 900 University Avenue, Riverside, California 92521; ‡ Department of Chemistry, University of California, Riverside, 900 University Avenue, Riverside, California 92521; † College of Horticulture and Plant Protection, Yangzhou University, Yangzhou 225009, China

**Keywords:** inducible promoter, heterologous gene expression, metabolomics, metabolic profiling, NMR

## Abstract

The filamentous fungus *Neurospora crassa* is a long-studied eukaryotic microbial system amenable to heterologous expression of native and foreign proteins. However, relatively few highly tunable promoters have been developed for this species. In this study, we compare the *tcu-1* and *nit-6* promoters for controlled expression of a GFP reporter gene in *N. crassa*. Although the copper-regulated *tcu-1* has been previously characterized, this is the first investigation exploring nitrogen-controlled *nit-6* for expression of heterologous genes in *N. crassa*. We determined that fragments corresponding to 1.5-kb fragments upstream of the *tcu-1* and *nit-6* open reading frames are needed for optimal repression and expression of GFP mRNA and protein. *nit-6* was repressed using concentrations of glutamine from 2 to 20 mM and induced in medium containing 0.5–20 mM nitrate as the nitrogen source. Highest levels of expression were achieved within 3 hr of induction for each promoter and GFP mRNA could not be detected within 1 hr after transfer to repressing conditions using the *nit-6* promoter. We also performed metabolic profiling experiments using proton NMR to identify changes in metabolite levels under inducing and repressing conditions for each promoter. The results demonstrate that conditions used to regulate *tcu-1* do not significantly change the primary metabolome and that the differences between inducing and repressing conditions for *nit-6* can be accounted for by growth under nitrate or glutamine as a nitrogen source. Our findings demonstrate that *nit-6* is a tunable promoter that joins *tcu-1* as a choice for regulation of gene expression in *N. crassa*.

Regulatable promoters are powerful tools for genetic analysis of protein functions in both prokaryotic and eukaryotic organisms. These promoters have been used with great advantage for analysis of the functions of essential genes (Richardson *et al.* 1989; Miyajima *et al.* 1987). A drawback to this strategy is the time needed to dilute the essential protein due to cell growth and turnover after repressing mRNA production from the promoter. Regulatable promoters have also been used to express/overexpress genes at a particular time during growth or development and to study the resulting phenotypes. In fungi, perhaps the best-characterized and most-used regulatable promoter is the divergent promoter that regulates expression of *GAL1* and *GAL10* in the yeast *Saccharomyces cerevisiae* (Matsumoto *et al.* 1981; Guarente *et al.* 1982; Johnston and Davis 1984). This promoter is repressed during growth on glucose, but induced in medium without glucose and containing galactose (Matsumoto *et al.* 1981; Guarente *et al.* 1982). The *GAL10* promoter has been used in a multitude of studies in yeast, including analysis of essential genes and regulated overexpression of genes (Richardson *et al.* 1989; Miyajima *et al.* 1987; Rose *et al.* 1987).

In recent years, there has been increasing interest in using microorganisms both for overexpression of native proteins and heterologous expression of proteins from other organisms (Medema *et al.* 2011). Examples of such applications in fungi are production of enzymes, pharmacologically active proteins, natural products, and biofuels (Cherry and Fidantsef 2003; Cary *et al.* 2012; Garvey *et al.* 2013; Kubicek *et al.* 2009; Shin and Yoo 2013). In many cases, constitutive promoters have been used to drive expression of homologous or heterologous proteins in fungi, with follow-up analysis of metabolite levels using NMR or mass spectrometry approaches (Anasontzis *et al.* 2014). However, for certain applications, regulatable promoters serve an important function in that they can be used to express toxic proteins or enzymes that produce metabolites that are themselves toxic to the cell (Scharf and Brakhage 2013). For example, a recent study in the filamentous fungus *Aspergillus nidulans* used the *alcA* promoter to achieve regulated expression of nonreducing polyketide synthases from *Aspergillus terreus*, with production of the expected products in good yield (Chiang *et al.* 2013).

*Neurospora crassa* is a model organism for the filamentous fungi, and available tools include a nearly complete gene knockout collection and more than 1000 mapped mutations (Perkins *et al.* 2001). To date, only a few regulatable promoters have been developed for use in *N. crassa*. The first such promoter, *qa-2*, is not highly induced and can only be turned on in low glucose (Campbell *et al.* 1994; Giles *et al.* 1985). *grg-1/ccg-1* is a glucose-repressible promoter (McNally and Free 1988) that has been used to drive expression of tyrosinase. However, the subsequent discovery that this promoter is also regulated by the circadian rhythm and blue light imposes additional requirements during harvesting of cells (Loros *et al.* 1989; Arpaia *et al.* 1995). Induction of the *cys-16* promoter requires growth on limiting sulfur (0.25 mM methionine), and this promoter has not been used to drive expression of heterologous genes (Reveal and Paietta 2012). The light-regulated *vvd* promoter is highly tunable but requires stringent control of lighting conditions during tissue collection (Hurley *et al.* 2012). The copper-regulated *tcu-1* promoter is also highly tunable and can operate in any genetic background (Lamb *et al.* 2013). The *nit-6* gene promoter is an alternative candidate for regulated protein expression in *N. crassa* (Lafferty and Garrett 1974; Prodouz and Garrett 1981). *nit-6* encodes NAD(P)H-nitrite reductase, the second step in nitrate assimilation (Lafferty and Garrett 1974; Prodouz and Garrett 1981). Expression of *nit-6* mRNA is controlled by nitrogen catabolite repression through the action of the GATA transcription factor NIT-2 (Exley *et al.* 1993; Fu and Marzluf 1990) and by nitrate-specific control mediated by the NIT-4 fungal binuclear cluster transcription factor (Exley *et al.* 1993; Fu *et al.* 1995). These two modes of regulation result in repression of *nit-6* during growth on glutamine or ammonium, but result in expression at high levels in medium containing nitrate as the sole nitrogen source (Exley *et al.* 1993).

In this study, we compare the *tcu-1* and *nit-6* promoters for regulated expression of genes in *N. crassa*. Although *tcu-1* has been previously characterized (Lamb *et al.* 2013), this is the first implementation of *nit-6* as a regulated promoter in *N. crassa*. We characterize the fragment sizes and conditions needed for induction and repression of these promoters. We also use proton (^1^H) NMR to perform metabolic profiling of cultures under inducing and repressing conditions for each promoter (Bundy *et al.* 2007; Barding *et al.* 2012; Larive *et al.* 2015). Our results provide a baseline for the metabolic changes that occur under the different growth conditions for each promoter.

## Materials and Methods

### Chemicals, media, and genetic procedures

Deuterium oxide (D_2_O, 99%) and ethylenediaminetetraacetic acid-*d_16_* (EDTA) were purchased from Cambridge Isotope Laboratories (Andover, MA). DSS-*d_6_* [3-(trimethylsilyl)-1-propanesulfonic acid sodium salt] was purchased from Isotec (Miamisburg, OH). Methanol-*d_4_* (CD_3_OD, 99.8%) and fungal protease inhibitor cocktail (#P8215) were obtained from Sigma-Aldrich (St. Louis, MO). Monobasic and dibasic sodium phosphate were purchased from Fisher Scientific (Pittsburgh, PA). TRIzol RNA Isolation Reagent (#15596-026) was purchased from Life Technologies (Grand Island, NY).

*N. crassa* strains are listed in Table 1. *N. crassa* hyphal cultures were propagated in Vogel’s minimal medium (VM) (Vogel 1964). VM without ammonium nitrate or containing sodium nitrate or glutamine (Gln) was used to induce or repress, respectively, the *nit-6* promoter (Exley *et al.* 1993). VM supplemented with 50 μM copper sulfate (Cu) was used to repress the *tcu-1* promoter, whereas VM containing bathocuproine disulphonate (BCS; #B1125-500MG; Sigma-Aldrich) was used to induce *tcu-1* (Korripally *et al.* 2010; Lamb *et al.* 2013). Sorbose-containing medium (FGS) (Davis and Deserres 1970) was used to facilitate colony formation on plates. *N. crassa* asexual spores (macroconidia or conidia) were propagated using standard procedures (Davis and Deserres 1970). *N. crassa* transformations were performed by electroporation using conidia as the recipient (Ivey *et al.* 1996). Sexual crosses were performed using standard procedures and ascospore progeny were plated on FGS medium (Davis and Deserres 1970). When indicated, modified VM or FGS medium (Pall 1993) contained the antibiotic phosphinothricin (purified as described previously) (Hays and Selker 2000) at 400 µg/ml. Pantothenate (pan) was used in media at 10 μg/ml.

**Table 1:** Strains used in this study

Strain	Relevant Genotype	Source/Reference
74-OR23-IVA	Wild type, *mat A*	FGSC2489
Δmus51-IV-8	Δ*rid-1*::*nat*, Δ*mus-51*::*nat*, *mat a*	FGSC23148; (S. Ouyang, I. E. Cabrera, A. J. Campbell, K. A. Borkovich, unpublished data)
pccg-1_GFP	Δ*pan-2*::*pccg-1-GFP-bar^R^*, *mat a*	This study
pnit-6_0.5	Δ*pan-2*::*pnit-6_0.5-GFP-bar^R^*, *mat a*	This study
pnit-6_1.0	Δ*pan-2*::*pnit-6_1.0-GFP-bar^R^*, *mat a*	This study
pnit-6_1.5	Δ*pan-2*::*pnit-6_1.5-GFP-bar^R^*, *mat a*	This study
ptcu-1_0.5	Δ*pan-2*::*ptcu-1_0.5-GFP-bar^R^*, *mat a*	This study
ptcu-1_1.0	Δ*pan-2*::*ptcu-1_1.0-GFP-bar^R^*, *mat a*	This study
ptcu-1_1.5	Δ*pan-2*::*ptcu-1_1.5-GFP-bar^R^*, *mat a*	This study

*N. crassa* liquid submerged cultures were inoculated with conidia to a final density of approximately 10^6^/ml in 50 ml of VM-pan liquid medium and incubated at 30° with shaking at 200 rpm for the indicated time in the dark. Tissue was collected on filter paper using a vacuum filtration system (EMD Millipore, Billerica, MA), flash-frozen in liquid nitrogen, and then pulverized in liquid nitrogen using a mortar and pestle.

### Construction of plasmid vectors and *N. crassa* strains

Two different groups of expression vectors were constructed during this study. Both groups of vectors target V5 and GFP-tagged open reading frames (ORFs) to the *pan-2* locus, creating a pantothenate auxotroph. However, the two vector groups implemented different promoters to drive expression of the V5-GFP fusion protein. One vector group contained the nitrogen-regulated *nit-6* (Exley *et al.* 1993) promoter (pRS426PVG/*pnit-6*), whereas the other contained the copper-regulated *tcu-1* (Lamb *et al.* 2013) promoter (pRS426PVG/*ptcu-1*). The first step of vector construction was to use yeast recombinational cloning (Colot *et al.* 2006) to replace the *ccg-1* promoter region in pRS426PVG (S. Ouyang, I. E. Cabrera, A. J. Campbell, K. A. Borkovich, unpublished data) with a fragment containing 0.5-, 1.0-, or 1.5-kb regions upstream of the *nit-6* or *tcu-1* genes. Then, two fragments were inserted into these modified vectors using yeast recombinational cloning. The first fragment included a multiple cloning sequence (MCS), a 5xGlycine linker, a V5-tag, and GFP sequence. The second fragment was the selectable marker gene *bar*, amplified from vector pTJK1 (Jones *et al.* 2007). *bar* confers resistance to phosphinothricin (Pall 1993). All fragments were amplified using Phusion High-Fidelity DNA Polymerase (New England Biolabs, Ipswich, MA). Primer sequences are listed in Table 2.

**Table 2:** Primer Sequences

Primer	Sequence (5’ to 3’)
Pan2-pnit6-1.5-FW	CCTTGCGTATATTCTGGACCGGTACAAGCTGGGAGCAGATGGAAAGACG
Pan2-pnit6-1.0-FW	CCTTGCGTATATTCTGGACCGGTACACGACATGCTAGCGACATCAATCC
Pan2-pnit6-0.5-FW	CCTTGCGTATATTCTGGACCGGTACTTACCATGGCCCGGTTTCCTAATCG
pnit6-RV	AACCCGGGGATCCACTAGTTCTAGATGCTGGCTGACGACAGAAAGACTAG
Pan2-ptcu1-1.5-FW	CCTTGCGTATATTCTGGACCGGTACGATGGGATAGAGAGAATGGCCGTTG
Pan2-ptcu1-1.0-FW	CCTTGCGTATATTCTGGACCGGTACGGTGAGCATGTTTTTGGCTTGGCTG
Pan2-ptcu1-0.5-FW	CCTTGCGTATATTCTGGACCGGTACACGGAACATCTCGTGAACAAGAAGG
ptcu1-RV	AACCCGGGGATCCACTAGTTCTAGAGGTTGGTTGGGGATGTGTGTGCG
Probe_GFP-FW	TGACCCTGAAGTTCATCTGC
Probe_GFP-RV	AACTCCAGCAGGACCATGTG

The pRS426PVG/*pnit-6* and pRS426PVG/*ptcu-1* vectors were separately transformed into recipient *N. crassa* strain Δmus51-IV-8 (S. Ouyang, I. E. Cabrera, A. J. Campbell, K. A. Borkovich, unpublished data) (Table 1), with selection on modified FGS medium plates containing phosphinothricin and pan. Strain Δmus51-IV-8 carries a mutation in the *rid* gene (Freitag *et al.* 2002), and is thus deficient in Repeat Induced Point mutation (RIP) (Selker 1990). RIP is a process that introduces mutations into duplicated DNA during a sexual cross in *N. crassa* (Selker 1990). It was necessary to include the Δ*rid* mutation to avoid mutating the *tcu-1* or *nit-6* promoters during crosses of the transformants to wild-type. Because *N. crassa* is multinucleate, most transformants are heterokaryons. Therefore, transformants were crossed to wild-type strain 74-OR23-IVA to isolate homokaryotic ascospore progeny. Ascospores were plated on FGS medium and strains were spot-tested on VM slants containing phosphinothricin and pan to identify resistant strains. Proper integration of the constructs at the *pan-2* locus was confirmed using diagnostic PCR with *pan-2* flanking region and gene-specific primers. Several progeny from the six crosses were tested for expression of GFP mRNA and protein (see below), with similar results. One representative strain was chosen for detailed analysis.

### Northern and Western blot analysis

Tissue from submerged cultures (see above) grown for 16 hr was used for isolation of total RNA for Northern blots and whole cell extracts were used for Western analysis. Total RNA was isolated using the TRIzol RNA Isolation Reagent following the manufacturer’s instructions. Samples containing 20 µg of total RNA were subjected to Northern analysis (Ouyang *et al.* 2014; Tsui *et al.* 1994). The GFP probe, corresponding to part of the gene ORF, was labeled as described previously (Kim and Borkovich 2004, 2006). ORF probes for *tcu-1* and *nit-6* were prepared in a similar manner and used to probe blots that were stripped after probing using GFP (Supporting Information, Figure S1). Gels were stained prior to transfer to use 18s-rRNA as a loading control for each blot. Whole cell extracts for Western analysis were obtained by adding buffer [50 mM Tris-Cl (pH 7.5), 0.5 mM EDTA, 0.5 mM phenylmethylsulfonyl fluoride and 0.1% fungal protease inhibitor cocktail] to pulverized hyphal mats. Samples were spun at 3300 rpm (1000×*g*) for 10 min at 4° in a microcentrifuge. The supernatant was retained and protein concentration was determined using the Bradford assay (Bio-Rad, Hercules, CA). Samples containing 50 µg of whole-cell extract protein were subjected to SDS-PAGE using 12.5% gels and gels transferred to nitrocellulose membranes as described previously (Krystofova and Borkovich 2005). Western analysis was performed using rabbit polyclonal GFP antibody (Cat. #ab290; Abcam, Cambridge, MA) as the primary antibody at a dilution of 1:2000. Horseradish peroxidase-conjugated Goat anti-Rabbit antibody (166-2408-MSDS; Bio-Rad) was used as the secondary antibody at a dilution of 1:5000. Chemiluminescent detection was performed using a Biochemi system (UVP, Upland, CA) described previously (Krystofova and Borkovich 2005).

### Cultures used for induction and repression of the *nit-6* and *tcu-1* promoters

For experiments involving the *nit-6* promoter, two identical sets of cultures were grown under repressing conditions (VM-Gln) for 14 hr. Cells were collected from each culture using a sterile filter paper/Buchner funnel assembly in a sterile hood and the cell pad was washed with sterile water. The cell pads were transferred to new flasks containing either VM-Gln (repressing conditions control) or VM-nitrate (inducing conditions) liquid medium. Cultures were grown for the indicated times (1–6 hr), after which cells were collected as described above and flash-frozen in liquid nitrogen.

For the *tcu-1* promoter, two sets of cultures were grown under repressing conditions (VM-Cu) for 14 hr. BCS (inducing conditions) was then added to a final concentration of 200 µM. An equal volume of water was used for the noninduced control. Both sets of cultures were then incubated with shaking for 3 hr. Cells were collected as described for the *nit-6* promoter (see above).

### Sample preparation for metabolomics experiments

Metabolite extractions were performed using a modification of a published procedure for *Fusarium* species (Lowe *et al.* 2010). Liquid cultures were harvested by vacuum filtration, washed three times in distilled water, and snap-frozen under liquid nitrogen. The material was ground using a mortar and pestle with liquid nitrogen and stored frozen at −80°. The frozen samples were freeze-dried on a lyophilizer overnight. A 50-mM phosphate buffer at pD 7.0 containing 0.05% (w/v) of each DSS-*d_6_* and EDTA-*d_16_* and composed of 80% D_2_O and 20% CD_3_OD was used for sample extraction. The value of pD was calculated using the pH meter reading (pH*) for a glass electrode calibrated with aqueous buffers using the equation pD = pH* + 0.4 to correct for the deuterium isotope effect (Glasoe and Long 1960). A sample containing 15 mg of dried material was resuspended in 1 ml of the extraction solvent and then heated at 50° for 10 min. After cooling, the samples were spun down in a microcentrifuge for 5 min. The supernatant was removed, transferred to a clean microcentrifuge tube, heated at 90° for 2 min, cooled to 4° for 45 min, and recentrifuged for 5 min. A 700-μl aliquot of the supernatant was transferred to a 5-mm NMR tube for ^1^H NMR.

### NMR measurements

^1^H NMR spectra were measured using a Bruker 600 MHz Avance spectrometer equipped with a triple gradient inverse probe operating at 599.58 MHz. Six samples of nit-6(Gln) and nit-6(nitrate) and five samples of tcu-1(Cu) and tcu-1(BCS) were examined in 5 mm NMR tubes. Spectra were recorded at 25° using presaturation for suppression of the residual water signal. A relaxation delay of 3.0 sec was used with an 8.50-μs 90° pulse at a power level of −5 dB. A spectral window of 7002.801 Hz was used, co-adding 256 scans into 42,014 complex points. Spectra were processed using Topspin 3.1 and zero-filled using 131,072 points with an applied 0.50 Hz line-broadening, with automated baseline correction to a fifth-order polynomial. Chemical shifts were referenced to DSS at 0.00 ppm. Resonance assignments were made by comparison to spectra measured for authentic standards and are consistent with previous metabolomics studies for *N. crassa* (Kim *et al.* 2011).

### Principal component analysis of NMR data

NMR spectra were exported from Topspin in ASCII file format and data pretreatment and principal component analysis (PCA) performed using Matlab R2013b, the PLS Toolbox 5.5, and m-files written in-house. Spectral regions containing the residual water resonance (4.575–5.075 ppm) below 0.5 and above 9.0 ppm were set as dark regions prior to PCA. To equalize the impact of variables in PCA, unit variance scaling was applied, which uses the SD of each variable as the scaling factor (van den Berg *et al.* 2006). After unit variance scaling, all metabolites have an SD of 1; therefore, the data are analyzed on the basis of correlations instead of covariance.

Visualization of loadings is challenging with unit variance–scaled models because the line shapes of loadings are not interpretable in the same way as in unscaled models and therefore cannot be used directly to identify peaks dominating the variance in the data. The PC loading (p) can be expressed as follows (Johnson and Wichern 1988),

$$p_{t_{i},r_{k}}=\frac{CC_{t_{i},r_{k}}\sqrt{\sigma_{r_{k}}}}{\sqrt{\lambda_{t_{i}}}}$$ (1)

where $\sigma_{r_{k}}$ and $\lambda_{t_{i}}$ are variances of spectral (peak intensity) variable $r_{k}$ and PC vector of scores $t_{i}$, respectively, and cc denotes the correlation coefficient between $r_{k}$ and $t_{i}$. In the unscaled case, the line shapes of PC loadings are interpretable because a loading value is proportional to the SD of a spectral variable σ_k_, which is, respectively, lower and higher for variables that constitute minor and major intensities of a peak. This interpretation is destroyed when each variable is scaled to unit variance, *i.e.*, σ_k_ = 1. To aid in the interpretation of the dominant source of the variance contribution in unit-variance PCA score plots, a loading value can be multiplied by the SD of its original spectral variable. Similar to the unscaled case, the line shapes of transformed (or scaled) unit variance loadings will be distorted, showing the first-derivative shape of a metabolite peak in cases in which the variation caused by variable peak position contributes to a model (Cloarec *et al.* 2005).

### Data availability

Strains and vectors are available upon request. The Supporting Information File contains detailed descriptions of all supplemental files.

## Results

### Construction of vectors and strains for regulated protein expression

A recent study demonstrated that a 1.5-kb region upstream of the *tcu-1* gene could function as a tunable, copper-regulated promoter to drive expression of essential genes in *N. crassa* (Lamb *et al.* 2013). An aim of this work was to determine whether shorter regions of the *tcu-1* upstream sequence could function as a promoter. We also explored the region upstream of the *nitrate-6* (*nit-6*) (Exley *et al.* 1993) gene for use as a regulated promoter for *N. crassa*. *nit-6* encodes the structural gene for nitrite reductase and is only expressed when *N. crassa* is cultured on medium containing nitrate and lacking a good nitrogen source (typically glutamine or ammonium) (Exley *et al.* 1993). The homologous promoter (*niiA*) has been demonstrated to be a tightly controlled promoter in the filamentous fungus *Aspergillus fumigatus* (Amaar and Moore 1998). We reasoned that *nit-6* might also be under tight regulation in *N. crassa* and would be useful for experiments requiring regulated protein expression.

**Figure 1:**
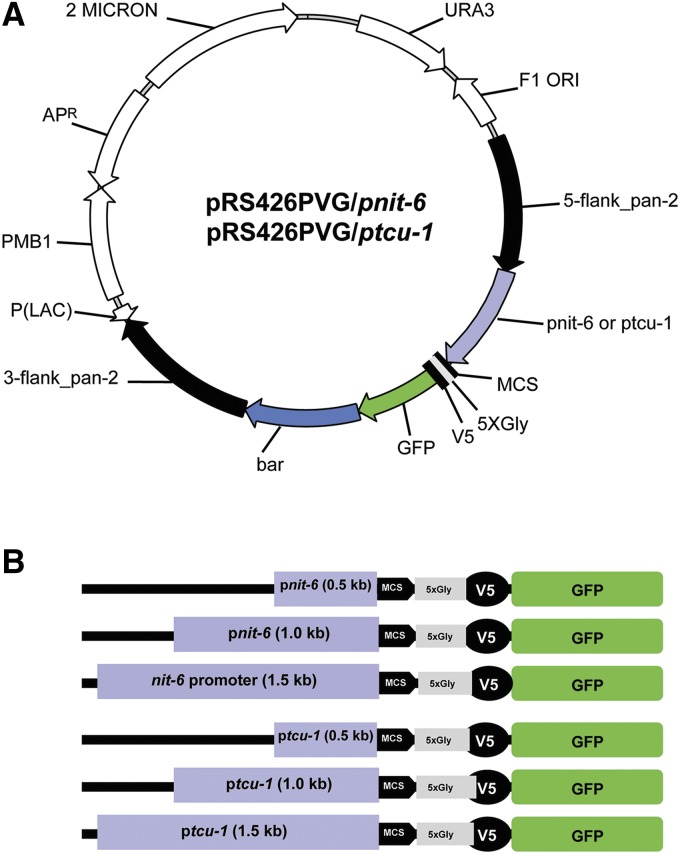
Vectors and promoter fragments. (A) Targeting/tagging backbone vectors pRS426PVG/pnit-6 and pRS426PVG/ptcu-1. Yeast/*E. coli* shuttle vector pRS426PVG (S. Ouyang, I. E. Cabrera, A. J. Campbell, K. A. Borkovich, unpublished data) is the backbone for both constructs. Both vectors confer uracil prototrophy to *ura3* yeast mutants (*URA3*) and ampicillin resistance (Ap^R^) in *E. coli*. pRS426PVG/ptcu-1 vectors contain fragments upstream of the *tcu-1* ORF, whereas the pRS426PVG/pnit-6 group of vectors contain *nit-6* upstream fragments. Both sets of vectors have the 5′ and 3′ flanking regions for the *N. crassa pan-2* ORF (black arrows) surrounding the *tcu-1* or *nit-6* promoter fragment (violet arrow), a multiple cloning sequence (MCS; black bar), a 5-glycine linker (5XGly; gray bar), a V5 peptide tag (V5-Tag; black bar), the GFP gene (GFP; green arrow) and the *bar* gene (blue shading), conferring resistance to phosphinothricin in *N. crassa* (*bar*). The 5′ *pan-2* flank extends from 1 kb upstream to the sequence just before the ATG, whereas the 3′ flank begins with the sequence just beyond the stop codon and extends 1 kb downstream. Other abbreviations: P(LAC), *lac* promoter; 2 MICRON, yeast 2 micrometer origin of replication; F1 ORI, origin of replication in *E. coli*. (B) Schematic representation of promoter fragments from *nit-6* and *tcu-1* cloned in the vectors. The regions 0.5, 1.0, and 1.5 kb upstream of the *tcu-1* or *nit-5* ORF were amplified using PCR and inserted into pRS426PVG using yeast recombinational cloning. The orientation of the promoter fragments relative to the MCS, 5XGly linker, V5 peptide tag, and GFP is shown.

We developed vectors for expression of proteins under control of regions corresponding to the *tcu-1* and *nit-6* promoters. The vectors were based on pRS426PVG (S. Ouyang, I. E. Cabrera, A. J. Campbell, K. A. Borkovich, unpublished data), developed to insert genes at the *pan-2* locus (Case and Giles 1958) in *N. crassa*. Mutation of *pan-2* leads to a requirement for pantothenic acid. The *ccg-1* promoter in pRS426PVG was replaced by fragments corresponding to upstream regions for the *nit-6* and *tcu-1* genes (Figure 1A). We amplified fragments corresponding to regions 0.5, 1.0, and 1.5 kb upstream of the start codon (Figure 1B). The *tcu-1* promoter group of vectors includes pRS426PVG/tcu-1_0.5 kb, pRS426PVG/tcu-1_1.0 kb, and pRS426PVG/tcu-1_1.5 kb, whereas the three vectors with the *nit-6* promoter fragments are pRS426PVG/pnit-6_0.5 kb, pRS426PVG/pnit-6_1.0 kb, and pRS426PVG/pnit-6_1.5 kb. All vectors contained a multiple cloning site, a 5-glycine linker, and V5 and GFP tags 3′ to the promoter fragment (Figure 1A). A *bar* cassette, conferring resistance to the antibiotic phosphinothricin (Pall 1993), was inserted 3′ to the GFP gene. The vectors were then transformed into the *N. crassa* recipient strain Δmus51-IV-8 (S. Ouyang, I. E. Cabrera, A. J. Campbell, K. A. Borkovich, unpublished data). A total of six strains were generated during this study (Table 1), each containing a different fragment of *tcu-1* or *nit-6* driving expression of GFP from the *pan-2* locus in *N. crassa*.

#### Expression of GFP from the different promoter fragments.

**Figure 2:**
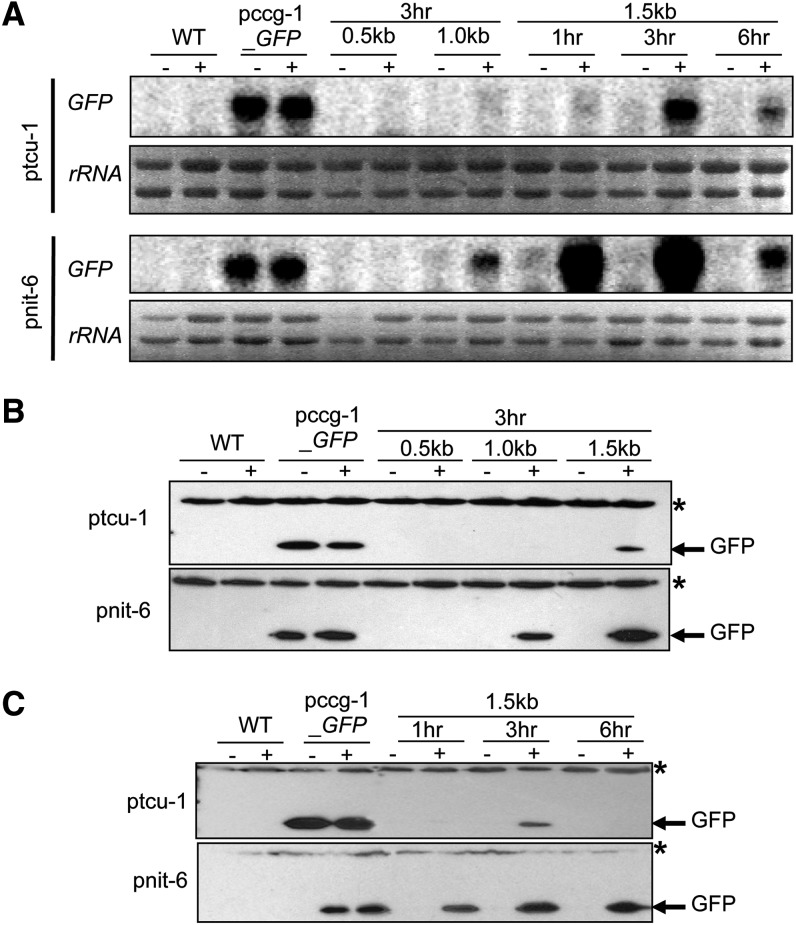
Identification of promoter fragments from *tcu-1* and *nit-6* that drive highest expression of GFP. (A) Northern analysis of GFP mRNA in the strains with different promoter fragments. Total RNA was isolated from strains cultured overnight in VM-Gln and then transferred to VM-nitrate medium for the indicated times. Samples containing 20 μg of total RNA were subjected to Northern analysis using GFP as a probe. 18s rRNA bands from the ethidium bromide-stained gel served as the loading control. Strains are wild-type 74-OR23-IVA (WT), pccg-1_GFP, pnit-6_0.5, pnit-6_1.0, pnit-6_1.5, ptcu-1_0.5, ptcu-1_1.0, and ptcu-1_1.5. The blot shown is representative of at least three experiments. (B) Western analysis of protein extracts from strains with different promoter fragments. Whole cell extracts were isolated from the strains with different promoter fragments and samples containing 50 μg total protein subjected to Western analysis using GFP antiserum (see *Materials and Methods* for details). The asterisk indicates a higher-molecular-weight, nonspecific background band. Strains are the same as in (A). The blot shown is representative of at least three experiments. (C) Time course expression of GFP proteins driven by 1.5-kb promoter fragments for *tcu-1* and *nit-6*. Whole cell extracts were isolated and subjected to Western analysis as described for (B). The asterisk indicates a nonspecific background band. Strains are wild-type 74-OR23-IVA (WT), pccg-1_GFP, ptcu-1_1.5, and pnit-6_1.5. Treatments: “-” refers to repressing conditions, which are VM-Gln for the pnit-6_1.5 strain and VM-Cu, for the ptcu-1_1.5 strain. “+” corresponds to inducing conditions, which are VM-nitrate for the pnit-6_1.5 strain and VM-BCS for the ptcu-1_1.5 strain. The blot shown is representative of at least three experiments.

We next investigated expression of GFP mRNA and protein in the six *N. crassa* strains carrying the different promoter fragments. For these studies, we used a strain in which the *ccg-1* promoter controls expression of GFP as a positive control (Figure 2A). The results showed that the growth regimens used for the different promoters did not influence expression of GFP mRNA or protein from *ccg-1* (Figure 2, A and B).

For experiments testing fragments from the 5′ upstream region of *tcu-1*, we grew *N. crassa* cells under repressing conditions (VM-Cu) and then added the copper chelator BCS to a final concentration of 200 µM (Lamb *et al.* 2013). The same volume of water served as a negative control. In initial experiments, cells were incubated for 3 hr after induction (Figure 2, A and B). Results from Northern and Western analysis showed that GFP mRNA and protein could only be detected in the tcu-1_1.5 strain and only after addition of BCS (Figure 2, A and B). The levels of GFP mRNA were comparable using the *ccg-1* and 1.5 kb *tcu-1* promoters, but protein levels were higher using *ccg-1* (Figure 2, B and C), perhaps suggesting some aspect of post-transcriptional control for the *tcu-1* driven Figuretranscript. Subsequent experiments using only the tcu-1_1.5 strain showed that GFP mRNA and protein levels peaked at 3 hr of induction and were greatly reduced at 6 hr (Figure 2, A and C). Thus, we concluded that use of the 1.5-kb promoter fragment and 3 hr of induction were sufficient to drive regulated expression of GFP under the *tcu-1* promoter in *N. crassa*. These results contrast with those from the previous study, in which induction of the *tcu-1* mRNA (not a fusion gene) peaked between 8 and 24 hr (Lamb *et al.* 2013). A possible explanation for this difference may be that GFP mRNA peaks faster but is less stable relative to *tcu-1* mRNA in *N. crassa*.

For the *nit-6* promoter fragments, we first cultured *N. crassa* with 20 mM Gln as the nitrogen source (repressing conditions), collected the cells, and transferred to fresh medium containing 20 mM sodium nitrate to induce expression for 3 hr (Exley *et al.* 1993). Northern blot analysis revealed that the GFP transcript could not be detected in the pnit-6_0.5 strain and that levels were very low in pnit-6_1.0 (Figure 2A). In contrast, GFP mRNA was abundantly expressed in cells carrying the 1.5-kb promoter fragment (strain pnit-6_1.5; Figure 2A). Western analysis showed that GFP protein could be detected using both the 1.0-kb and 1.5-kb promoter fragments, with higher levels using the 1.5-kb promoter (Figure 2B). Levels of GFP mRNA were higher using the 1.5-kb *nit-6* promoter fragment than with *ccg-1* (Figure 2A), whereas GFP protein levels exhibited a less dramatic difference (Figure 2B). Later experiments with the pnit-6_1.5 strain showed that levels of GFP mRNA were elevated at 1 hr of induction, increased slightly at 3 hr, and then greatly diminished at 6 hr (Figure 2A). Less of a difference was observed in the amount of GFP protein produced under these conditions, with significant levels detected at time points 1 hr, 3 hr, and 6 hr, and with the greatest amount observed after 3 hr and 6 hr of induction (Figure 2C). These experiments suggested that the 1.5-kb fragment was necessary to promote significant expression of heterologous genes using the *nit-6* promoter and that mRNA and protein levels peaked by 3 hr after induction. In addition, GFP mRNA and protein levels were higher in experiments using the 1.5-kb *nit-6* promoter than the 1.5-kb *tcu-1* promoter (Figure 2).

We compared the induction profile of GFP driven by the 1.5-kb *tcu-1* and *nit-6* promoter fragments to that of the endogenous *tcu-1* and *nit-6* ORFs by stripping and reprobing the blots used in Figure 2 with *tcu-1* or *nit-6* ORF probes (Figure S1). The results demonstrated that *tcu-1* mRNA could be detected after 1 hr, increasing to much higher levels at 3 hr, and then greatly decreasing at 6 hr after addition of BCS to the medium (Figure S1A, top panel). An identical pattern of expression was observed for the endogenous *nit-6* gene (Figure S1A, bottom panel). These results showed that expression of the GFP reporter mirrors that of the endogenous *tcu-1* gene. The timing of expression of the native *nit-6* gene was similar to that of GFP, but levels of GFP mRNA were greater than those of *nit-6* at 1 hr.

### Regulation of the nit-6 promoter by nitrate and glutamine

We further explored the conditions needed for induction and repression of the 1.5-kb *nit-6* promoter fragment. Initial experiments examined the concentration of nitrate needed to induce expression after long-term growth in 20 mM Gln. After overnight growth in VM-Gln, cells from strain pnit-6_1.5 were transferred to VM containing 0.5–30 mM sodium nitrate (Figure S2). Cultures were incubated for an additional 3 hr and total RNA and protein extracts were isolated as described above. Northern blot results indicated that GFP mRNA levels were similar under all concentrations of sodium nitrate (Figure S2). This result indicates that the *nit-6* promoter is extremely sensitive to nitrate, with full induction of mRNA at 0.5 mM. Results from Western blot analysis of protein extracts isolated from the same tissue revealed that GFP levels increased slightly with higher concentrations of sodium nitrate, peaking at ∼20 mM nitrate (Figure S2).

**Figure 3:**
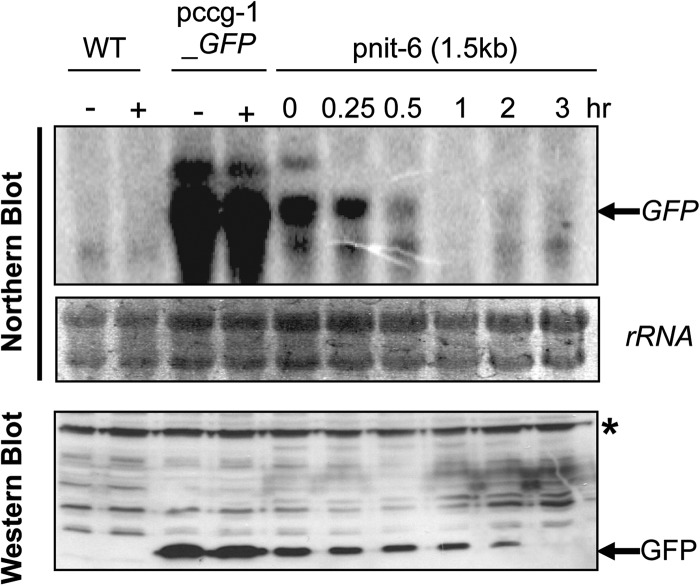
Repression of the 1.5 kb *nit-6* promoter fragment by Gln. Strains were cultured in VM-nitrate medium overnight and then transferred to VM-Gln (20 mM glutamine) for the indicated amounts of time (0.25–3 hr). Northern and Western analysis were performed using total RNA and protein extracts isolated as described for Figure 2. The asterisk indicates a nonspecific background band. Strains are wild-type 74-OR23-IVA (WT), pccg-1_GFP, and ptcu-1_1.5. Treatments are the same as in Figure 2. The blot shown is representative of at least three experiments.

We next investigated the kinetics of repression of the *nit-6* promoter by Gln after long-term growth in nitrate. Levels of GFP mRNA and protein were examined in cultures grown in nitrate (inducing conditions) for 14 hr and then transferred to medium containing 20 mM Gln for different time periods (Figure 3). The strain with the *ccg-1* promoter was used as a control. The results demonstrated that levels of GFP transcript are lower using the *nit-6* promoter (inducing conditions) *vs.* *ccg-1* after overnight growth. These findings are consistent with those showing that GFP transcript levels peak at 3 hr and then are greatly reduced at 6 hr after induction (Figure 2A). GFP transcript levels were decreased approximately two-fold within 30 min after transfer to glutamine and GFP mRNA could not be detected after 1 hr (Figure 3). In contrast, GFP protein levels declined more gradually, decreasing at all time points until protein could not be detected (3 hr; Figure 3). Of course, the relative stability of different proteins will determine how rapidly they are cleared from the cell after the transfer to repressing conditions.

We compared the repression of the endogenous *nit-6* gene by glutamine to that of GFP by stripping and reprobing the blot used in Figure 3. The results showed that expression of *nit-6* is greatly reduced after 15 min of exposure to glutamine, with the mRNA not detectable by 1 hr of treatment (Figure S1B). This repression profile is similar to that observed for GFP driven by the 1.5 kb *nit-6* promoter fragment (Figure 3). Taken together, these results suggest that *nit-6* is a viable promoter to use for experiments involving both induction and repression of genes in *N. crassa*.

### ^1^H NMR metabolite profiles

**Figure 4:**
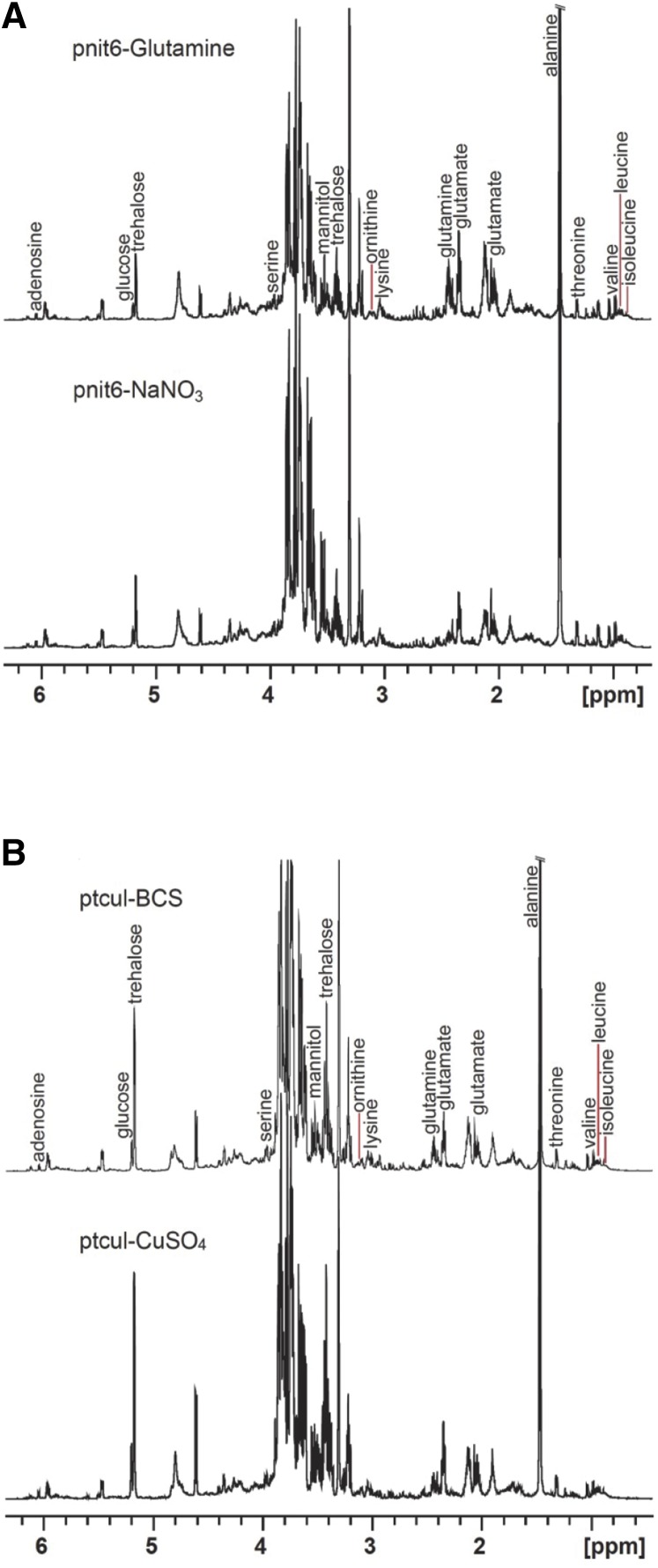
Representative ^1^H NMR spectra with key resonances labeled. Although the crowded region between 3.59 and 3.92 ppm contains the resonances of several metabolites, the predominant peaks in this region are those of trehalose. (A) Spectra of extracts of the pnit-6_1.5 strain cultured on VM-Gln (top) or VM-nitrate (bottom). The resonances of Gln and Glu are increased when Gln is used as a nitrogen source, whereas the levels of Ala, mannitol, and trehalose are more intense when the strain is grown on nitrate-containing media (see also Figure S3 and Figure S4). (B) Spectra of extracts of the ptcu-1_1.5 strain cultured on media containing in BCS (top) and CuSO_4_ (bottom). These spectra are more similar than those shown in (A), with the most pronounced differences in the regions containing the resonances of BCS (see also Figure S5).

^1^H NMR provides an untargeted view of metabolic shifts resulting from expression of the inserted gene. Figure 4 presents representative ^1^H NMR spectra for each sample type with key resonances labeled. We were not surprised that use of Gln as a nitrogen source (Figure 4A) significantly increases the levels of Gln and glutamate (Glu) compared with the expression on media containing sodium nitrate. In contrast, the resonances of trehalose and mannitol are lower in the extracts of pnit-6_1.5 grown on Gln. The levels of alanine (Ala) also decrease in the pnit-6_1.5(Gln) samples, which can be more readily observed in the expansion of the spectra presented in Figure S3 at a lower vertical scale. Taken together, the lower levels of trehalose, Ala, and mannitol suggest a shift away from glycolytic metabolism compared to growth of the pnit-6_1.5 promoter strain on VM-nitrate (Dijkema *et al.* 1985). Expansions of NMR spectra measured for biological replicates (Figure S4A [pnit-6_1.5(Gln)] and Figure S4B [pnit-6_1.5(nitrate)]) demonstrate that the cell culture and extraction methods are highly reproducible and allow for a more detailed evaluation of the impact of culture conditions on the metabolite profile. It is important to note that the differences in the metabolic profiles for pnit-6_1.5(Gln) and pnit-6_1.5(nitrate) largely reflect the different growth conditions required to repress or induce the *nit-6* promoter, and not the products of the expressed gene. This is confirmed by comparison of the ^1^H NMR spectra for the wild-type *N. crassa* strain 74-OR23-IVA (Table 1) grown on Gln and nitrate (Figure S5), with the results presented in Figure S3 for pnit-6_1.5.

In contrast, comparison of the spectra shown in Figure 4B reveal that the use of the copper expression trigger causes truly minimal perturbation of the *N. crassa* metabolic profile. The major difference between the NMR spectra measured for the five replicate samples of ptcu-1_1.5 strain grown on medium containing BCS (Figure S6A) or on CuSO_4_-containing medium (Figure S6B) is the presence of the BCS resonances, highlighted in blue.

### PCA analysis

Although obvious differences can be discerned by visual inspection of the ^1^H NMR spectra for the different samples and treatments, PCA provides an unbiased global analysis of sample variance (Ramadan *et al.* 2006; Worley and Powers 2013). PCA is an unsupervised technique, meaning that the analysis is performed independent of variable identification. Unit variance scaling was used in our calculations to reduce the bias that can be introduced for the most intense signals. In this study, the PCA score plots indicate the extent to which individual ^1^H NMR spectra within a treatment group are similar and the spectra between treatment groups differ. Although statistical significance cannot be deduced from groupings in PCA scores plots, sample groupings do provide insights into differences between samples (Barding *et al.* 2012).

**Figure 5:**
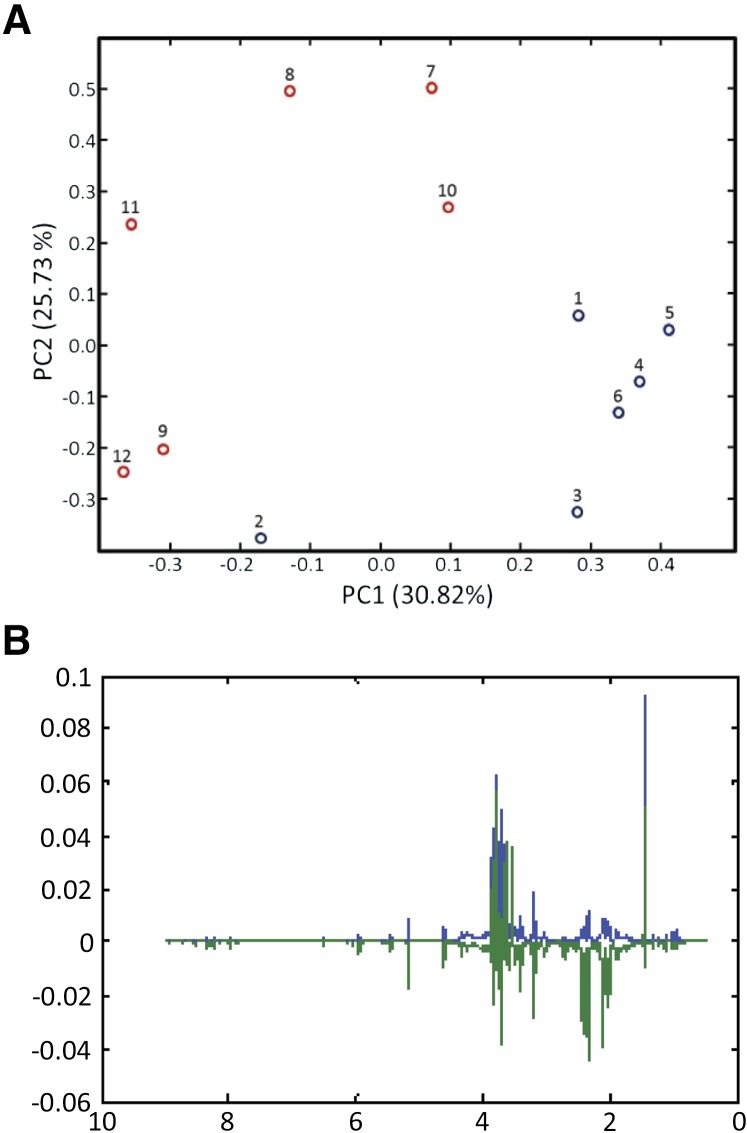
PCA results for the ^1^H NMR spectra of strain pnit-6_1.5 cultured on Gln (samples 1–6; blue) and nitrate (samples 7–12; red). (A) PCA scores. Although the samples within a treatment are not tightly clustered, clear segregation of the samples by treatments is observed in the scores plot. (B) PCA loadings. The loadings plot highlights the ^1^H NMR resonances that make the greatest contributions to sample variance in this data set. The loadings for PC1 are shown in blue, and those for PC2 are shown in green.

A clustering of the samples by treatment type is observed in the PCA score plots for the pnit-6_1.5(Gln) and pnit-6_1.5(nitrate) samples (Figure 5A). Together, PC1 and PC2 account for 57% of the variance in this sample set. Although the samples are not tightly grouped, there is clear segregation of the samples of pnit-6_1.5 grown on VM-Gln (samples 1–6) and grown on media containing nitrate (samples 7–12). The PCA loadings highlight the variables, in this case metabolite NMR resonances, responsible for the greatest variance for a particular principal component (*e.g.*, PC1 or PC2). Analysis of the loadings plot (Figure 5B) confirms our observations that the levels of Gln, Glu, Ala, mannitol, and trehalose differ significantly in the NMR spectra measured for the two treatments. In addition, the PCA loadings highlight the resonances between 0.5 and 1.5 ppm due to amino acids (*e.g.*, valine, leucine, isoleucine, and threonine) that we did not identify as important in our analysis of the NMR spectra. Although the intensity of the PCA loading for a particular variable is an indicator of its relative importance, it should be recognized that loadings reflect the variance over all samples and cannot necessarily be assigned to individual treatments. This is especially the case when, as in Figure 5A, the sample groupings separate along both PC1 and PC2. In addition, in interpreting these results it should be noted that the signs of the loadings are arbitrary and are not correlated to an increase or decrease in resonance intensity.

**Figure 6:**
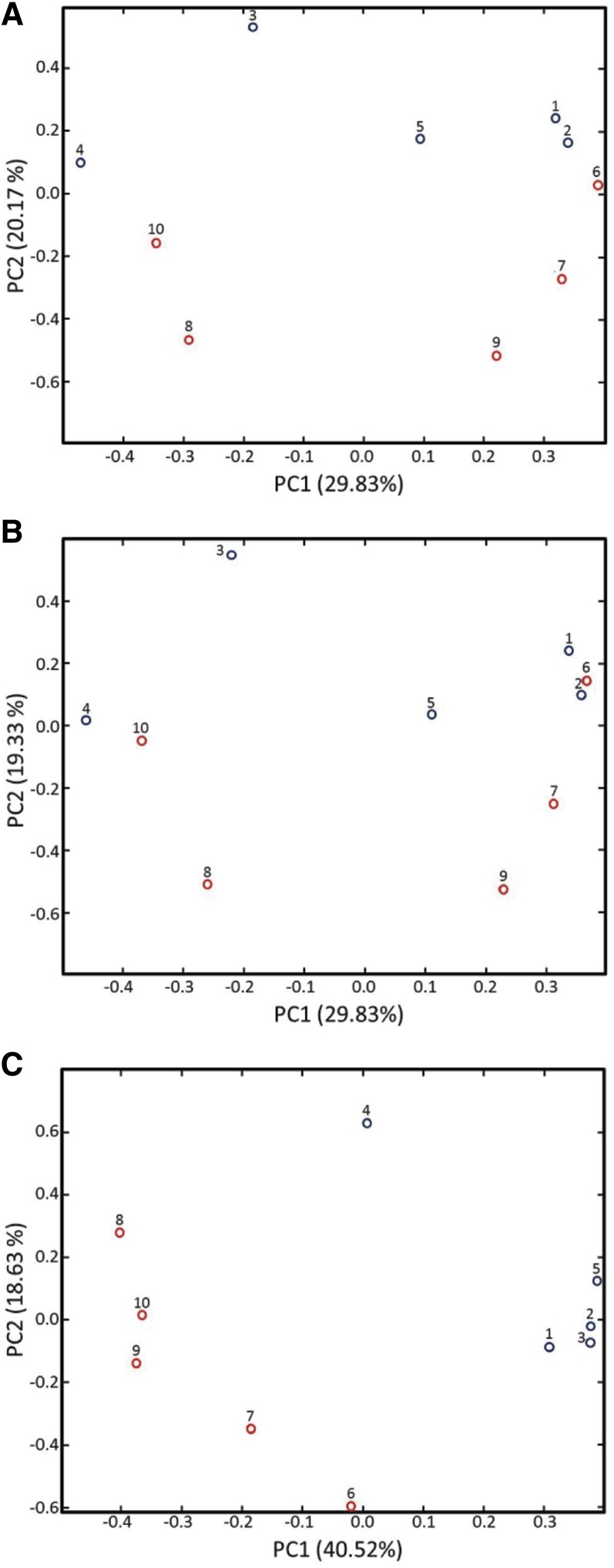
PCA scores plots comparing the ^1^H NMR spectra of ptcu-1_1.5 grown on media containing BCS (samples 1–5; blue) and CuSO_4_ (samples 6–10; red). (A) Scores plot calculated using the full NMR spectra. Sample segregation by treatment is observed along PC2 with the ptcu-1_1.5 (BCS) samples appearing at the top of the plot. (B) Scores plot for spectra with the regions containing the BCS resonances removed. Removal of the BCS resonances reduces the degree of segregation by treatment. (C) Scores plot calculated using only the spectral regions containing the BCS resonances. Performing the analysis using only the regions of the spectrum that contain the BCS peaks increases the segregation of the samples by treatment and indicates that the BCS peaks are primarily responsible for the sample segregation in (A), as confirmed by the loadings (data not shown).

Figure 6 presents the PCA results for the ptcu-1_1.5(BCS) and ptcu-1_1.5(Cu) samples. In Figure 6A the scores were calculated using all the variables, *i.e.*, the full ^1^H NMR spectra except for the dark regions, which included the residual water resonance (4.575–5.075 ppm) and baseline (below 0.5 and above 9.0 ppm). Although the most variance is accounted for by PC1 (29.83%), the segregation of the sample groupings is observed along PC2. Examination of the loadings (data not shown) indicated the BCS resonances were the largest factor in the sample grouping in Figure 6, consistent with our visual observations of the NMR spectra for these samples. To further test this idea, the dataset was modified by removing the regions of the ^1^H NMR spectra containing the BCS resonances and PCA was performed again. The results of this analysis (Figure 6B) show a reduction in the extent of segregation of the samples compared with the scores plot in Figure 6A. In contrast, if the analysis is performed using only the regions of the spectra containing the BCS resonances (Figure 6C), the segregation of the samples by treatment is increased. Taken together, these results confirm that the ptcu-1_1.5 copper expression trigger results in a minimal metabolic perturbation as reflected by ^1^H NMR.

## Discussion

In this study, we develop *nit-6* as an inducible/repressible promoter for *N. crassa*. We demonstrate that a region corresponding to 1.5 kb upstream of the *nit-6* ORF achieves tight repression by glutamine and induction by nitrate. We show that the same sized region is required for regulation of the recently described *tcu-1* promoter (Lamb *et al.* 2013). We compare *nit-6* to *tcu-1* with regard to the amplitude and timing of induction of a GFP reporter. The results showed that *nit-6* leads to higher levels of mRNA and protein than *tcu-1*, but with similar kinetics. Levels of GFP message and protein were comparable using the *ccg-1* promoter and inducing conditions for the *nit-6* promoter. Furthermore, the timing of expression of GFP was similar to that of the endogenous *tcu-1* and *nit-6* genes in the same strains.

By testing promoter fragments corresponding to 0.5, 1.0, and 1.5 kb upstream of the *tcu-1* and *nit-6* ORFs, we determined that 0.5 and 1.0 kb fragments were insufficient to drive high levels of expression of GFP mRNA and protein. The results for *tcu-1* can be explained by the large 5′ untranslated region (UTR) present on the mRNA (657 nts; Broad Institute *Neurospora crassa* Database; http://www.broadinstitute.org/annotation/genome/neurospora/MultiHome.html); use of the 1-kb fragment would only leave 343 nts for a promoter. In the case of *nit-6*, the Broad Database shows a relatively small 193-nt UTR. However, an *in silico* analysis of the upstream region of *nit-6* (Chiang and Marzluf 1995) revealed three possible NIT-4 binding sites at −695, −677, and −547 and eight GATA elements at −713, −445, and −235. The authors did not report whether they searched further upstream for additional NIT-2 binding sites, but these results suggest that at least 713 nts upstream of the ORF would be necessary for proper regulation of *nit-6*.

The impact of the growth conditions used for promoter induction on *N. crassa* metabolism was evaluated using ^1^H NMR, a well-established technique for metabolic profiling (Larive *et al.* 2015). The metabolites detected are summarized in Table S1. For both the pnit-6_1.5 and ptcu-1_1.5 strains, no metabolic perturbation was detected as a direct result of GFP expression. The growth conditions used for induction of *tcu-1* are truly minimally perturbing, as detected by ^1^H NMR. Although the different growth conditions used for induction/repression of *nit-6* produced minor (and anticipated) changes in the levels of some primary metabolites, the metabolic response was similar to that observed for the wild-type.

There are several examples of carbon-regulated promoters used in fungi, including the *GAL1*/*GAL10* promoter in *S. cerevisiae* (Matsumoto *et al.* 1981; Guarente *et al.* 1982; Johnston and Davis 1984) and *alcA* in *A. nidulans* (Waring *et al.* 1989). Both of these promoters are repressed during growth on glucose and induced using either galactose (*GAL1/GAL10*) (Matsumoto *et al.* 1981; Guarente *et al.* 1982; Johnston and Davis 1984) or ethanol/threonine (*alcA*) (Waring *et al.* 1989; Bailey and Arst 1975). Relevant to use of carbon-regulated promoters in fungi and effects on the metabolome, we previously analyzed the metabolic profile of *N. crassa* after growth on medium containing high (1.5%) or limiting (0.15%) glucose (Kim *et al.* 2011). Our results demonstrated that carbon availability influences the global metabolic profile in wild-type *N. crassa*, with differences in levels of several amino acids, intracellular glucose, mannitol, and other compounds (Kim *et al.* 2011). Our results in this study using nitrate *vs.* Gln for regulation of *nit-6* demonstrated that *tcu-1* causes minimal perturbation of the metabolome and would be most useful for applications whereby maintenance of a consistent metabolic state is required. We also anticipate that the use of metabolic profiling in conjunction with gene expression will be increasingly important in future studies in which the expression products have a significant metabolic impact because of their direct or indirect effect on specific metabolite pathways or because of unanticipated toxic effects.

The *A. fumigatus niiA* promoter (Amaar and Moore 1998), homologous to *nit-6*, has been used successfully to regulate gene expression in this organism, including that of essential genes (Hu *et al.* 2007). In both *N. crassa* and *A. fumigatus*, *nit-6/niiA* is tightly regulated by nitrogen source. This is crucial for study of essential gene functions and for expression of potentially toxic proteins from heterologous sources. Our work demonstrates the metabolic consequences of using this promoter, which may warrant consideration for certain applications, particularly those involving expression of genes in nitrogen-regulated pathways in both *A. fumigatus* and *N. crassa*.

In summary, our findings demonstrate that *nit-6* is a tunable promoter that joins *tcu-1* as an option for regulated expression of genes in *N. crassa*. In addition, ^1^H NMR metabolic profiling proved to be useful in assessing the impacts of the inserted promoters on the levels of primary metabolites and identifying changes that resulted from differences in the growth conditions used to induce or repress expression.

## Supplementary Material

Supporting Information
