# Supplementary material for: Metabolic Impacts of Using Nitrogen and Copper-Regulated Promoters to Regulate Gene Expression in Neurospora crassa
Source: G3 (Bethesda). 2015 Jul 20;5(9):1899–908. doi: 10.1534/g3.115.020073 (PMC4555226; doi:10.1534/g3.115.020073)
Supplement: Supporting Information [file supp_5_9_1899__index.html]

Metabolic Impacts of Using Nitrogen and Copper-Regulated Promoters to Regulate Gene Expression in Neurospora crassa — Supporting Information 

# Metabolic Impacts of Using Nitrogen and Copper-Regulated Promoters to Regulate Gene Expression in *Neurospora crassa*

## Supporting Information for Ouyang *et al.*, 2015

**Files in this Data Supplement:**

- Supporting Information - Table S1 and Figures S1-S6 (PDF, 1 MB)
- Table S1 - *N. crassa* metabolites detected by 1H NMR. (PDF, 74 KB)
- Figure S1 - Regulation of the endogenous *tcu-1* and *nit-6* mRNAs. (PDF, 161 KB)
- Figure S2 - Derepression of GFP mRNA and protein levels after exposure to different nitrate concentrations. (PDF, 199 KB)
- Figure S3 - Expansion of the spectra of pnit-6\_1.5 cultured on Gln (top) and nitrate (bottom) at lower vertical scale to highlight differences in the intensity the Ala resonance. (PDF, 135 KB)
- Figure S4 - Overlays of the 1H NMR spectra for all six replicates of strain pnit-6\_1.5. (PDF, 476 KB)
- Figure S5 - Expansion of the spectra of wild-type *N. crassa* (WT) cultured on Gln (top) and nitrate (bottom). (PDF, 128 KB)
- Figure S6 - Overlays of 1H NMR spectra for all five replicates of strain ptcu-1\_1.5. (PDF, 471 KB)
