# Supplementary material for: Metabolic Impacts of Using Nitrogen and Copper-Regulated Promoters to Regulate Gene Expression in Neurospora crassa
Source: G3 (Bethesda). 2015 Jul 20;5(9):1899–908. doi: 10.1534/g3.115.020073 (PMC4555226; doi:10.1534/g3.115.020073)
Supplement: Supporting Information [file supp_g3.115.020073_020073SI.pdf]

G3 Genes | Genomes | Genetics

SUPPORTING INFORMATION

Metabolic impacts of using nitrogen and copper-regulated promoters to regulate gene expression in *Neurospora crassa*

Shouqiang Ouyang[1,3], Consuelo N. Beecher[2], Kang Wang [2], Cynthia K. Larive[2*] and Katherine A. Borkovich[1,*]

[1]Department of Plant Pathology and Microbiology

[2]Department of Chemistry

University of California, Riverside

900 University Avenue

Riverside, CA 92521

[3]College of Horticulture and Plant Protection

Yangzhou University

Yangzhou 225009, China

*Co-Corresponding Authors

Email: katherine.borkovich@ucr.edu

Email: clarive@ucr.edu

DOI: 10.1534/g3.115.020073

Table S1. *N. crassa* metabolites detected by ^{1}H NMR. Metabolite resonances were identified by comparison of the spectra of extracts with those of authentic standards measured under similar conditions. The metabolite marked with an asterisk was tentatively identified.

ATP
adenosine
alanine
arginine
carnitine
glucose
glucose-1-phosphate*
glutamate
glutamine
isoleucine
lactate
leucine
lysine
mannitol
ornithine
phenylalanine
serine
threonine
trehalose
tyrosine
UDP-galactose
UDP-glucose
UDP-*N*-acetylgalactosamine
UDP-*N*-acetylglucosamine

Figure S1

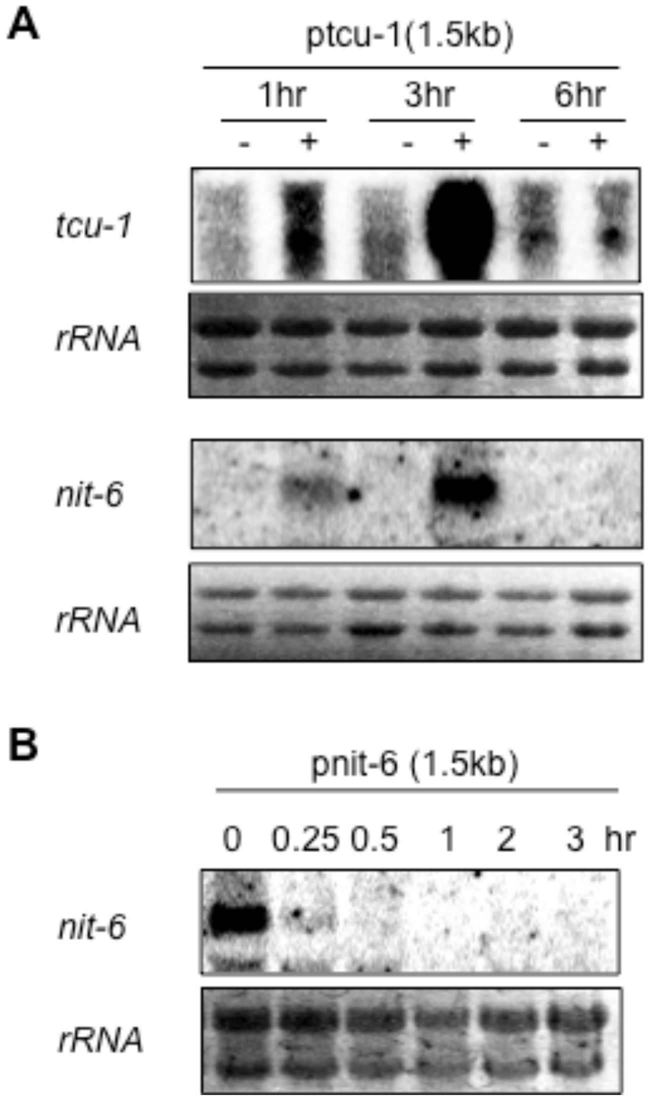

Figure S1. Regulation of the endogenous *tcu-1* and *nit-6* mRNAs. A. Induction profiles for *tcu-1* and *nit-6* by BCS and nitrate, respectively. The blots from Fig. 2 were stripped and reprobed with probes corresponding to the ORF for *tcu-1* (top panel) or *nit-6* (bottom panel). The corresponding lanes for the rRNA control blots from Fig. 2 are shown for comparison. **B. Repression of the endogenous *nit-6* gene by glutamine.** The blot from Fig. 3 was stripped and reprobed with an ORF probe for *nit-6*. The corresponding lanes for the rRNA control blot from Fig. 3 is shown for comparison.

Figure S2

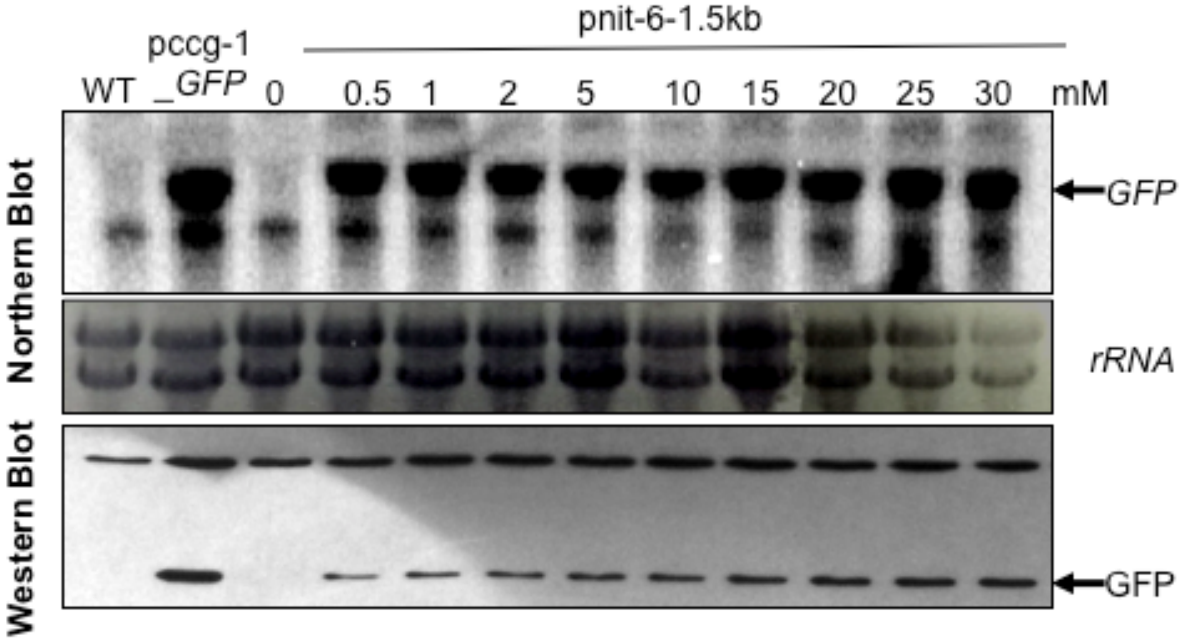

Figure S2. Derepression of GFP mRNA and protein levels after exposure to different nitrate concentrations. Strains were cultured in VM-Gln medium overnight and then transferred to VM containing the indicated concentrations of nitrate. Total RNA and protein were analyzed for GFP mRNA and protein levels using northern and western analysis as described in the legend to Fig. 2. Strains are wild type 74-OR23-IVA (WT), pccg-1_GFP and pnit-6_1.5.

Figure S3

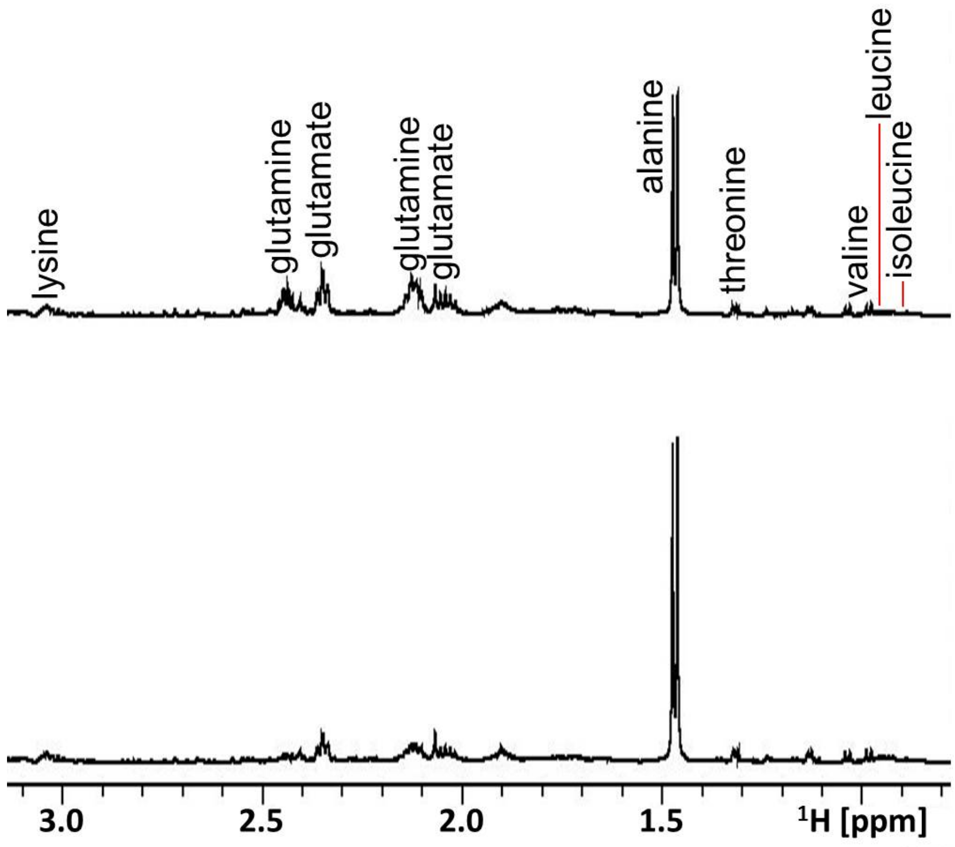

Figure S3. Expansion of the spectra of pnit-6_1.5 cultured on Gln (top) and nitrate (bottom) at lower vertical scale to highlight differences in the intensity the Ala resonance. The increase in the intensity of the Gln and Glu resonances can also be clearly seen in the top spectrum.

Figure S4

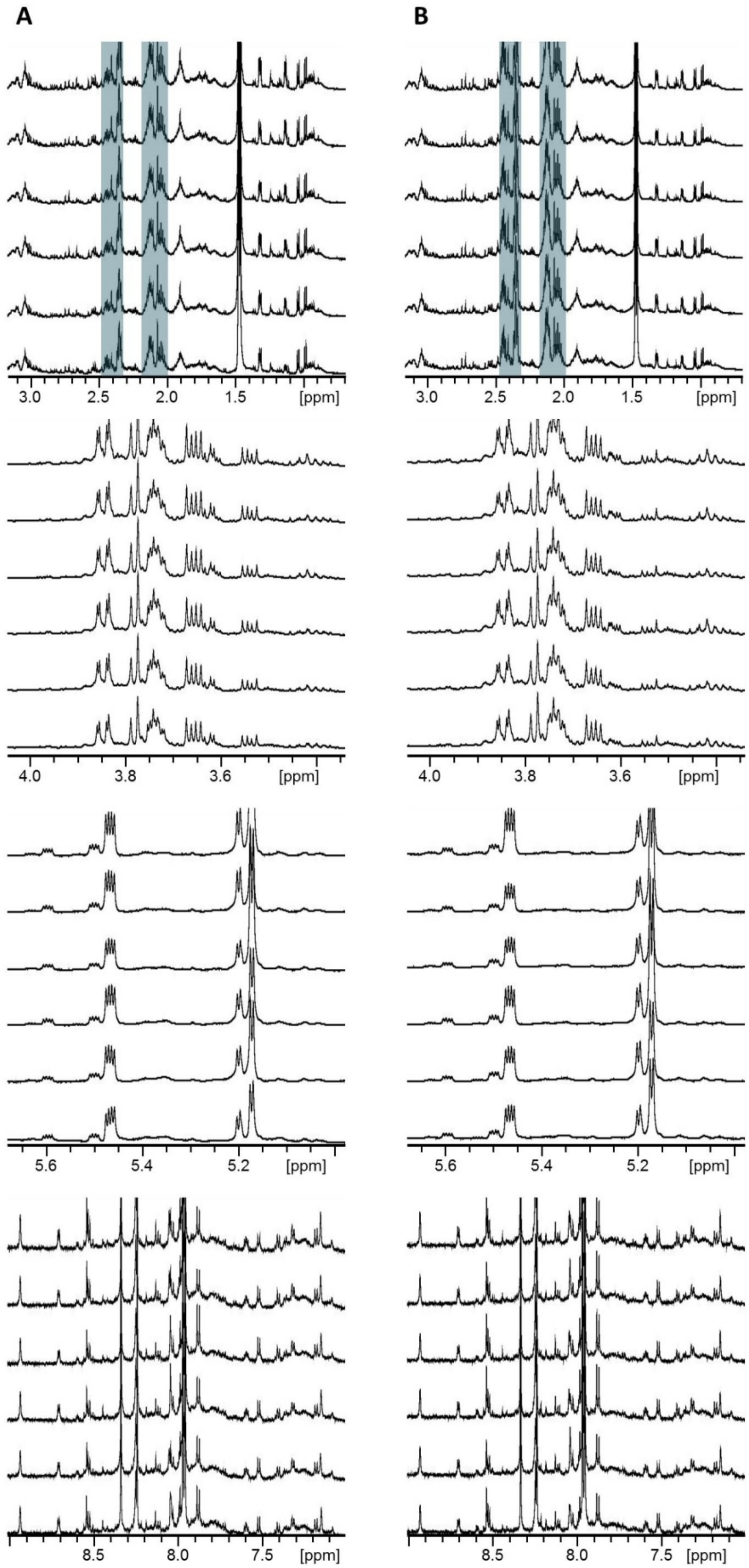

Figure S4. Overlays of the ^{1}H NMR spectra for all six replicates of strain pnit-6_1.5. The resonances of Gln and Glu are highlighted in blue. Each spectral region is scaled to compensate for differences in resonance intensity.

A. Spectra measured for biological replicates of pnit-6_1.5 cultured on VM-nitrate.

B. Spectra measured for biological replicates of pnit-6_1.5 cultured on VM-Gln. The resonances of Gln and Glu are significantly more intense in these spectra than in **A**.

Figure S5

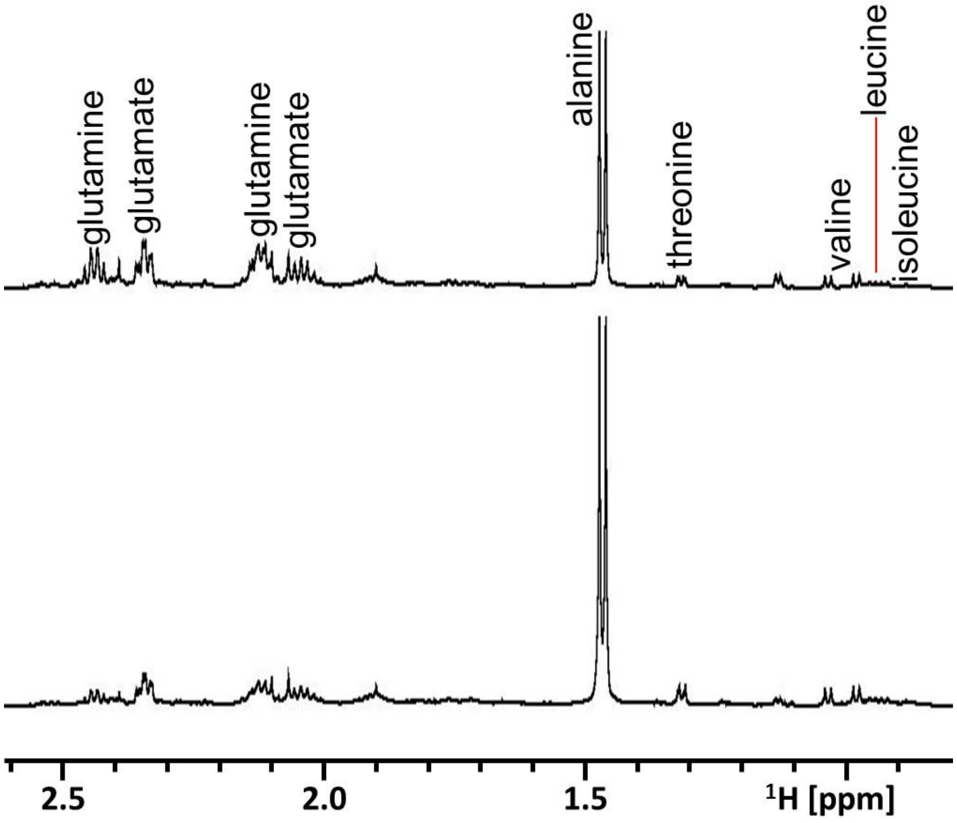

Figure S5. Expansion of the spectra of wild-type *N. crassa* (WT) cultured on Gln (top) and nitrate (bottom. The different growth conditions produce similar metabolic profiles for wild type strain 74-OR23-IVA as were observed for pnit-6_1.5 (Fig. S3); namely higher levels of Gln and Glu and lower levels of Ala when grown using glutamine as the nitrogen source.

Figure S6

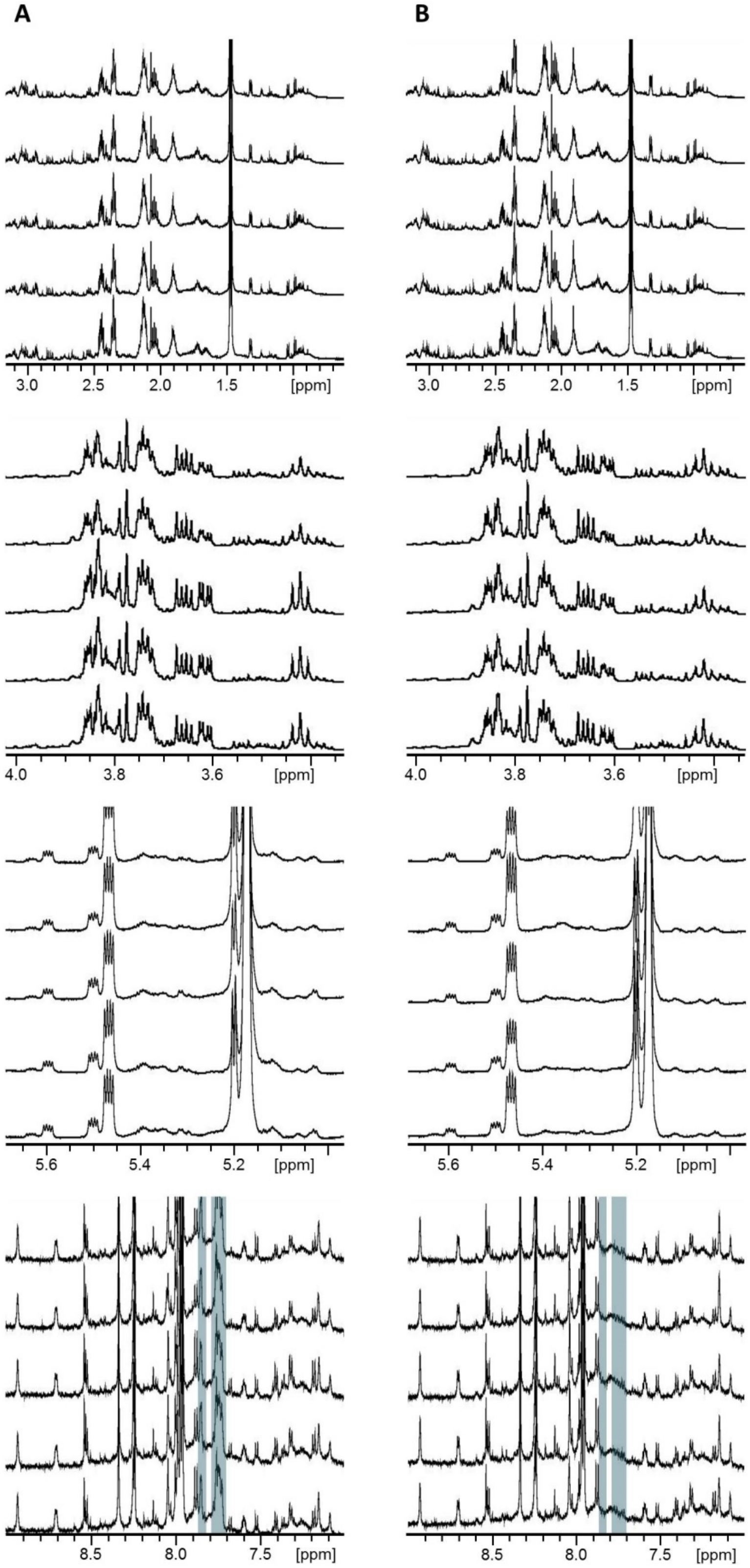
A
B
3.0
2.5
2.0
1.5
[ppm]
3.0
2.5
2.0
1.5
[ppm]
4.0
3.8
3.6
[ppm]
4.0
3.8
3.6
[ppm]
5.6
5.4
5.2
[ppm]
5.6
5.4
5.2
[ppm]
8.5
8.0
7.5
[ppm]
8.5
8.0
7.5
[ppm]

Figure S6. Overlays of ^{1}H NMR spectra for all five replicates of strain ptcu-1_1.5. The region of the spectra containing the BCS resonances are highlighted in blue. Each spectral region is scaled to compensate for differences in resonance intensity.

A. Spectra measured for biological replicates of ptcu-1_1.5 cultured on VM-BCS.

B. Spectra measured for biological replicates of pnit-6_1.5 cultured on VM-Cu. Note the absence of the BCS resonances observed between 7.7 and 7.9 ppm in **A**.
