# Supplementary material for: Metabolic Impacts of Using Nitrogen and Copper-Regulated Promoters to Regulate Gene Expression in Neurospora crassa
Source: G3 (Bethesda). 2015 Jul 20;5(9):1899–908. doi: 10.1534/g3.115.020073 (PMC4555226; doi:10.1534/g3.115.020073)
Supplement: Supporting Information [file supp_g3.115.020073_TableS1.pdf]

Table S1. *N. crassa* metabolites detected by ^{1}H NMR. Metabolite resonances were identified by comparison of the spectra of extracts with those of authentic standards measured under similar conditions. The metabolite marked with an asterisk was tentatively identified.

ATP
adenosine
alanine
arginine
carnitine
glucose
glucose-1-phosphate*
glutamate
glutamine
isoleucine
lactate
leucine
lysine
mannitol
ornithine
phenylalanine
serine
threonine
trehalose
tyrosine
UDP-galactose
UDP-glucose
UDP-*N*-acetylgalactosamine
UDP-*N*-acetylglucosamine
