# Supplementary material for: Metabolic Impacts of Using Nitrogen and Copper-Regulated Promoters to Regulate Gene Expression in Neurospora crassa
Source: G3 (Bethesda). 2015 Jul 20;5(9):1899–908. doi: 10.1534/g3.115.020073 (PMC4555226; doi:10.1534/g3.115.020073)
Supplement: Supporting Information [file supp_g3.115.020073_FigureS1.pdf]

Figure S1

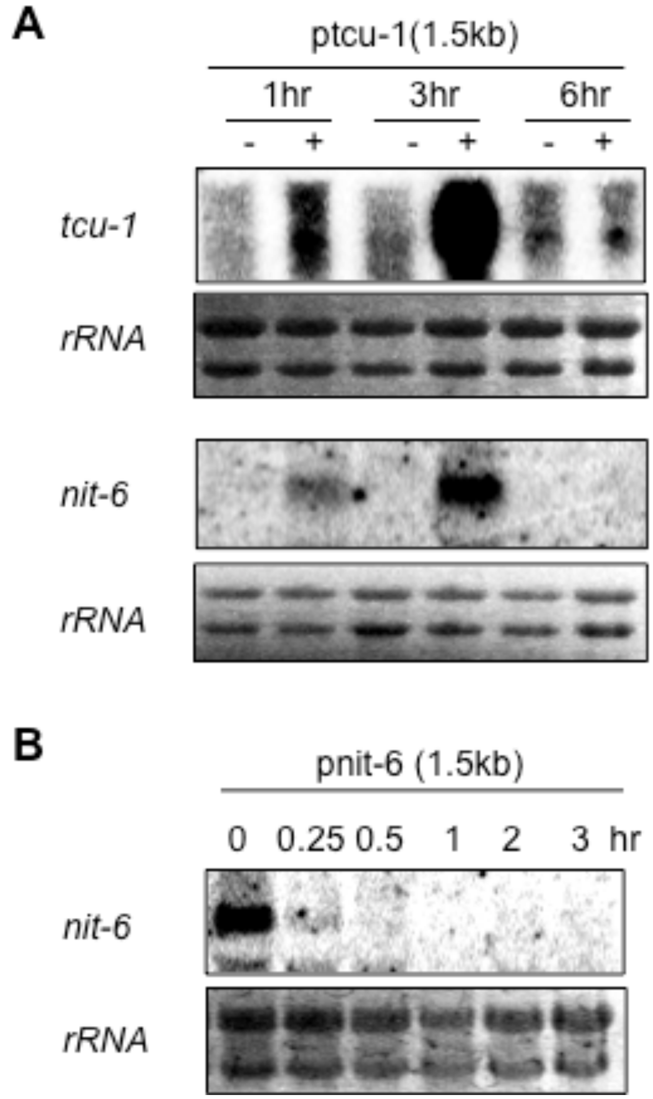

Figure S1. Regulation of the endogenous *tcu-1* and *nit-6* mRNAs. A. Induction profiles for *tcu-1* and *nit-6* by BCS and nitrate, respectively. The blots from Fig. 2 were stripped and reprobed with probes corresponding to the ORF for *tcu-1* (top panel) or *nit-6* (bottom panel). The corresponding lanes for the rRNA control blots from Fig. 2 are shown for comparison. **B. Repression of the endogenous *nit-6* gene by glutamine.** The blot from Fig. 3 was stripped and reprobed with an ORF probe for *nit-6*. The corresponding lanes for the rRNA control blot from Fig. 3 is shown for comparison.
