# Supplementary material for: Metabolic Impacts of Using Nitrogen and Copper-Regulated Promoters to Regulate Gene Expression in Neurospora crassa
Source: G3 (Bethesda). 2015 Jul 20;5(9):1899–908. doi: 10.1534/g3.115.020073 (PMC4555226; doi:10.1534/g3.115.020073)
Supplement: Supporting Information [file supp_g3.115.020073_FigureS2.pdf]

Figure S2

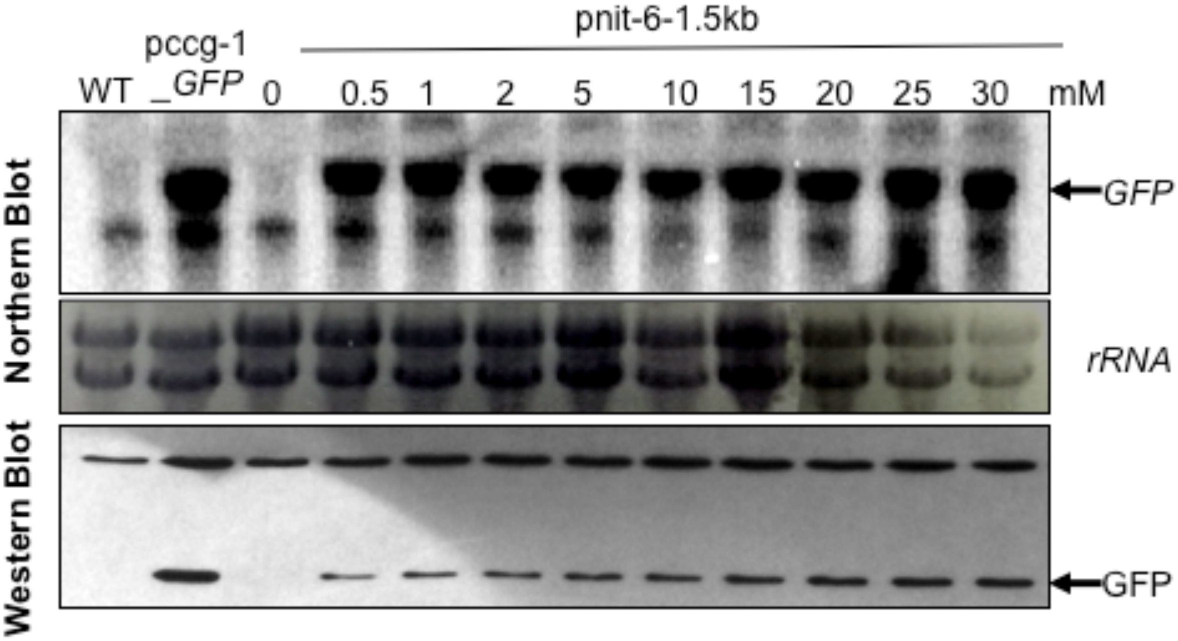

Figure S2. Derepression of GFP mRNA and protein levels after exposure to different nitrate concentrations. Strains were cultured in VM-Gln medium overnight and then transferred to VM containing the indicated concentrations of nitrate. Total RNA and protein were analyzed for GFP mRNA and protein levels using northern and western analysis as described in the legend to Fig. 2. Strains are wild type 74-OR23-IVA (WT), pccg-1_GFP and pnit-6_1.5.
