# Supplementary material for: Metabolic Impacts of Using Nitrogen and Copper-Regulated Promoters to Regulate Gene Expression in Neurospora crassa
Source: G3 (Bethesda). 2015 Jul 20;5(9):1899–908. doi: 10.1534/g3.115.020073 (PMC4555226; doi:10.1534/g3.115.020073)
Supplement: Supporting Information [file supp_g3.115.020073_FigureS3.pdf]

Figure S3

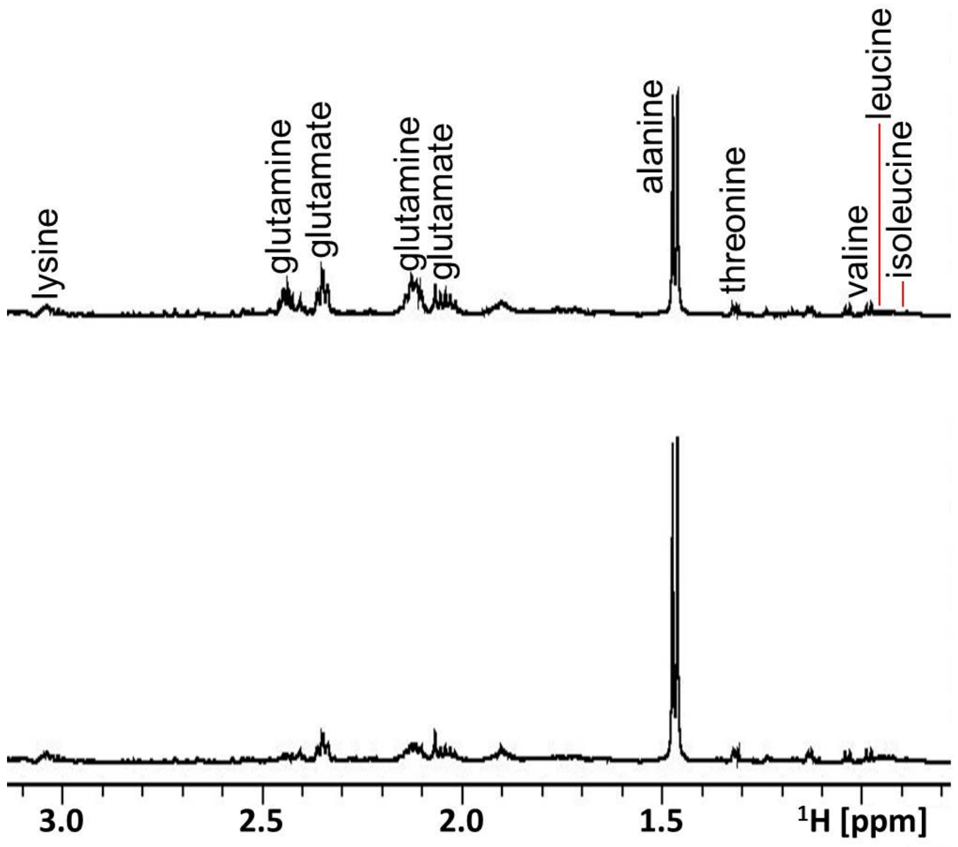

Figure S3. Expansion of the spectra of pnit-6_1.5 cultured on Gln (top) and nitrate (bottom) at lower vertical scale to highlight differences in the intensity the Ala resonance. The increase in the intensity of the Gln and Glu resonances can also be clearly seen in the top spectrum.
