# Supplementary material for: Metabolic Impacts of Using Nitrogen and Copper-Regulated Promoters to Regulate Gene Expression in Neurospora crassa
Source: G3 (Bethesda). 2015 Jul 20;5(9):1899–908. doi: 10.1534/g3.115.020073 (PMC4555226; doi:10.1534/g3.115.020073)
Supplement: Supporting Information [file supp_g3.115.020073_FigureS4.pdf]

Figure S4

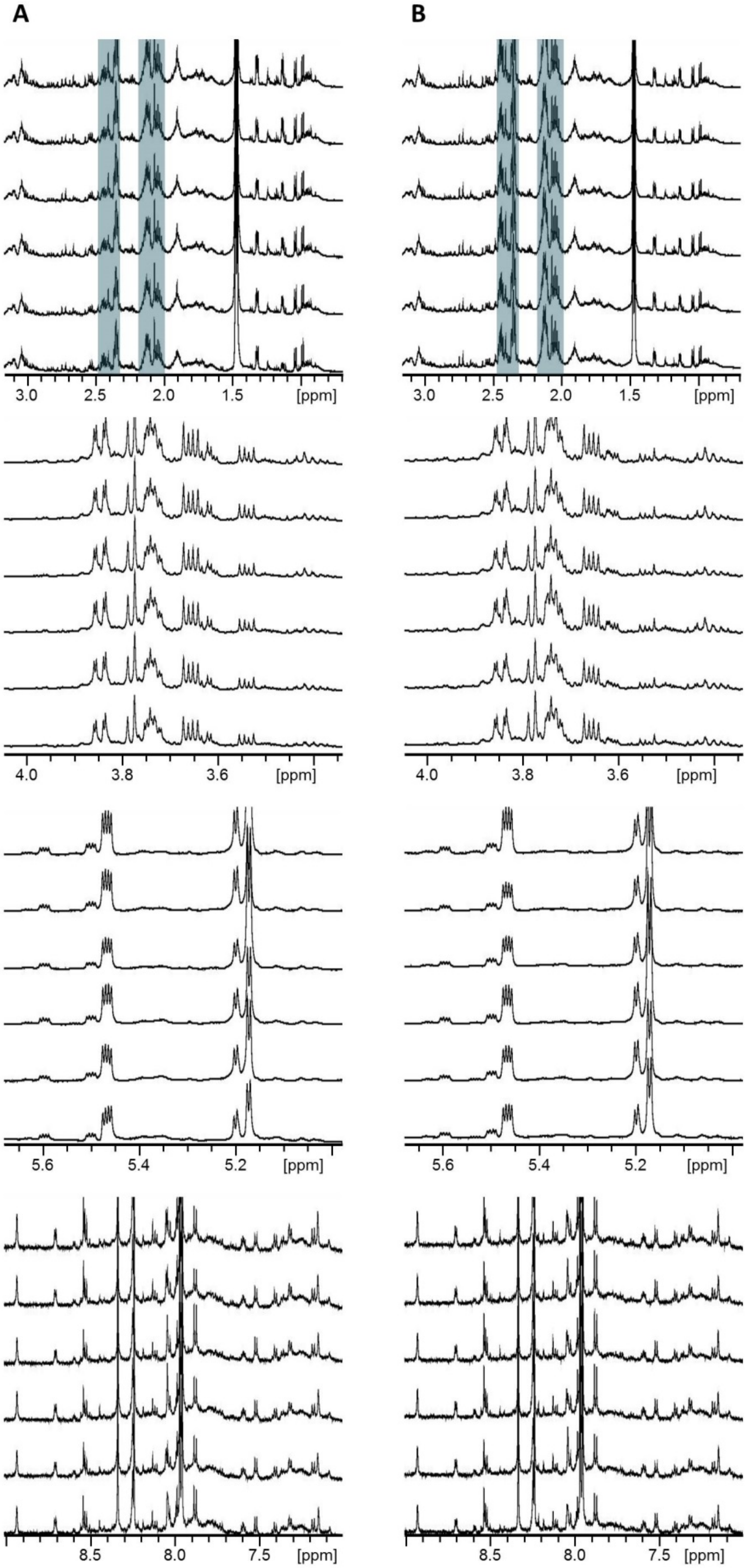

Figure S4. Overlays of the ^{1}H NMR spectra for all six replicates of strain pnit-6_1.5. The resonances of Gln and Glu are highlighted in blue. Each spectral region is scaled to compensate for differences in resonance intensity.

A. Spectra measured for biological replicates of pnit-6_1.5 cultured on VM-nitrate.

B. Spectra measured for biological replicates of pnit-6_1.5 cultured on VM-Gln. The resonances of Gln and Glu are significantly more intense in these spectra than in **A**.
