# Supplementary material for: Metabolic Impacts of Using Nitrogen and Copper-Regulated Promoters to Regulate Gene Expression in Neurospora crassa
Source: G3 (Bethesda). 2015 Jul 20;5(9):1899–908. doi: 10.1534/g3.115.020073 (PMC4555226; doi:10.1534/g3.115.020073)
Supplement: Supporting Information [file supp_g3.115.020073_FigureS5.pdf]

Figure S5

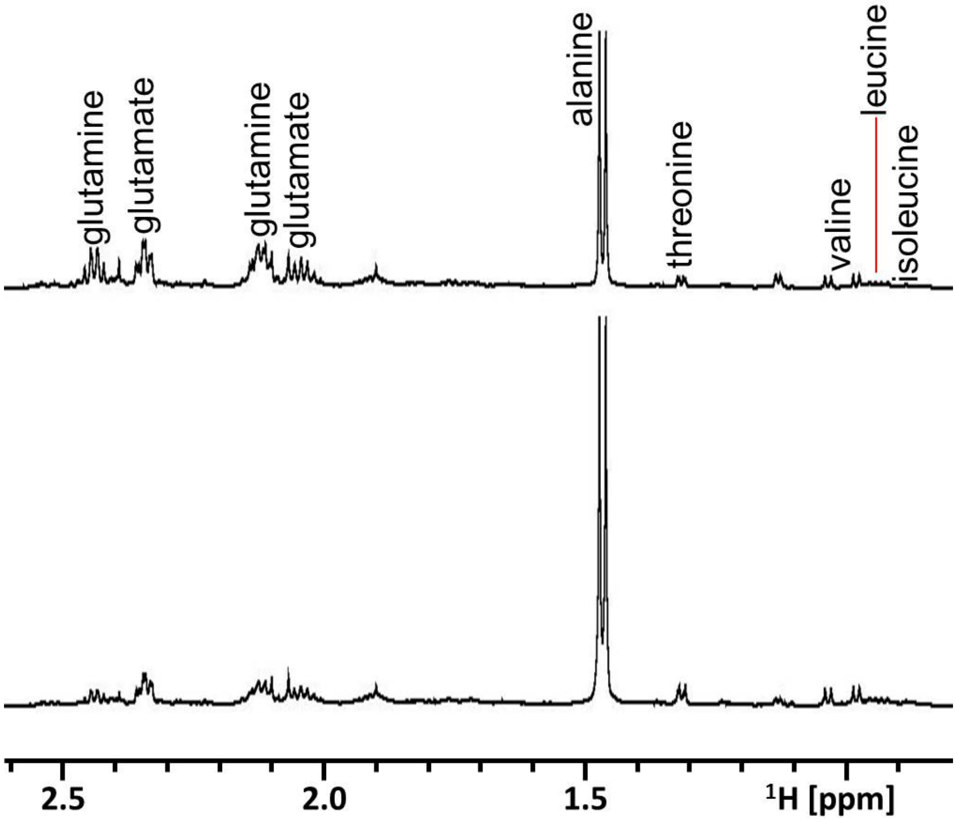

Figure S5. Expansion of the spectra of wild-type *N. crassa* (WT) cultured on Gln (top) and nitrate (bottom. The different growth conditions produce similar metabolic profiles for wild type strain 74-OR23-IVA as were observed for pnit-6_1.5 (Fig. S3); namely higher levels of Gln and Glu and lower levels of Ala when grown using glutamine as the nitrogen source.
