# Supplementary material for: Metabolic Impacts of Using Nitrogen and Copper-Regulated Promoters to Regulate Gene Expression in Neurospora crassa
Source: G3 (Bethesda). 2015 Jul 20;5(9):1899–908. doi: 10.1534/g3.115.020073 (PMC4555226; doi:10.1534/g3.115.020073)
Supplement: Supporting Information [file supp_g3.115.020073_FigureS6.pdf]

## Figure S6

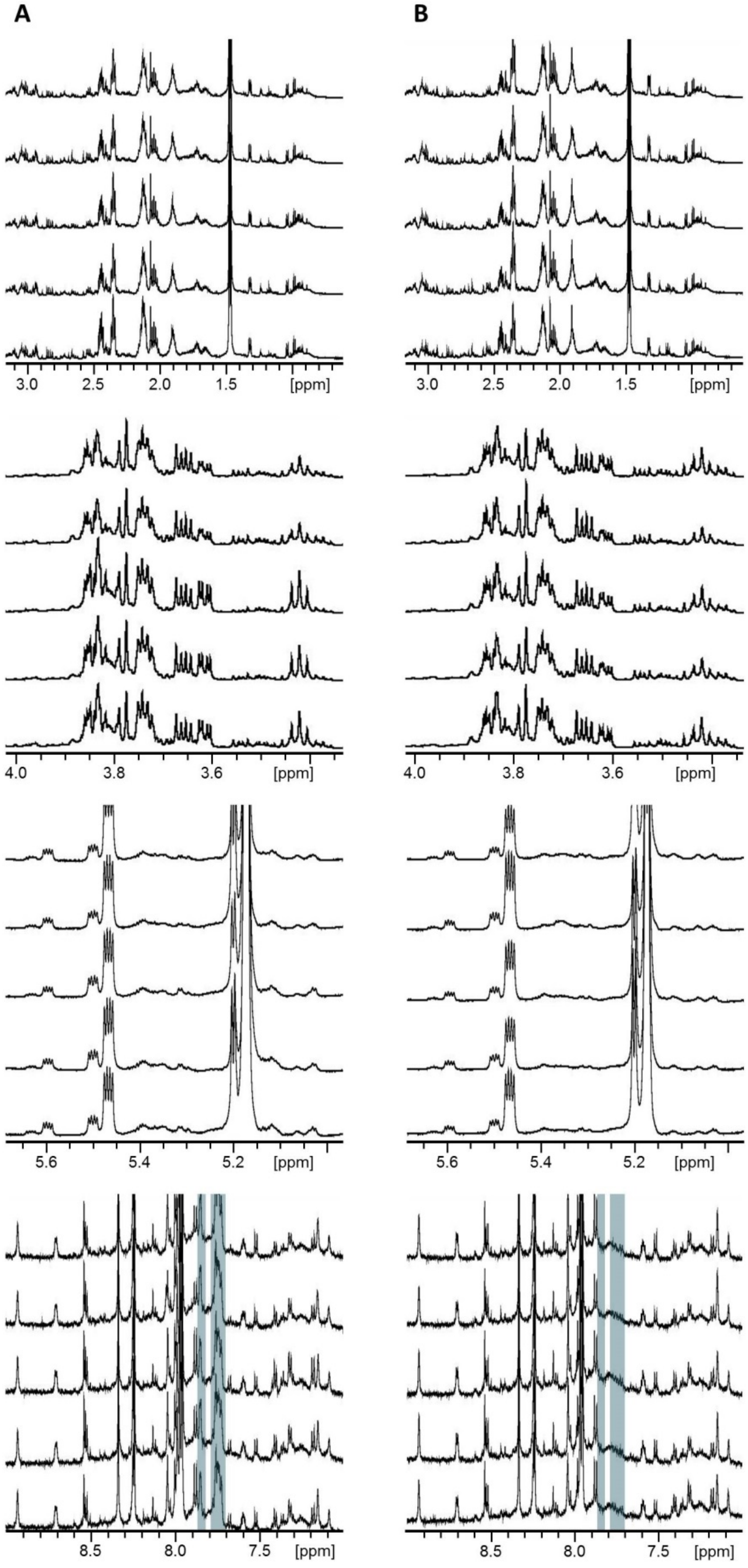

**Figure S6. Overlays of $^1$H NMR spectra for all five replicates of strain ptcu-1_1.5.** The region of the spectra containing the BCS resonances are highlighted in blue. Each spectral region is scaled to compensate for differences in resonance intensity.

**A. Spectra measured for biological replicates of ptcu-1_1.5 cultured on VM-BCS.**

**B. Spectra measured for biological replicates of pnit-6_1.5 cultured on VM-Cu.** Note the absence of the BCS resonances observed between 7.7 and 7.9 ppm in **A**.
